# Impact of retrotransposon protein L1 ORF1p expression on oncogenic pathways in hepatocellular carcinoma: the role of cytoplasmic PIN1 upregulation

**DOI:** 10.1038/s41416-023-02154-9

**Published:** 2023-01-27

**Authors:** Bassier Zadran, Praveen Dhondurao Sudhindar, Daniel Wainwright, Yvonne Bury, Saimir Luli, Rachel Howarth, Misti Vanette McCain, Robyn Watson, Hannah Huet, Fanni Palinkas, Rolando Berlinguer-Palmini, John Casement, Derek A. Mann, Fiona Oakley, John Lunec, Helen Reeves, Geoffrey J. Faulkner, Ruchi Shukla

**Affiliations:** 1 Newcastle University Centre for Cancer, Biosciences Institute, Faculty of Medical Sciences, Newcastle University, Newcastle-upon-Tyne, NE2 4HH UK; 2 Department of Cellular Pathology, Newcastle upon Tyne Hospitals NHS Foundation Trust, Newcastle-upon-Tyne, UK; 3 Newcastle University Centre for Cancer, Clinical and Translational Research Institute, Faculty of Medical Sciences, Newcastle University, Newcastle-upon-Tyne, NE2 4HH UK; 4 Bioimaging Unit, Newcastle University, Newcastle-upon-Tyne, NE2 4HH UK; 5 Bioinformatics Support Unit, Newcastle University, Newcastle-upon-Tyne, NE2 4HH UK; 6 Department of Gastroenterology and Hepatology, School of Medicine, Koç University, Istanbul, Turkey; 7 Hepatopancreatobiliary Multidisciplinary Team, Freeman Hospital, Newcastle-upon-Tyne Hospitals NHS foundation, Newcastle-upon-Tyne, UK; 8 Mater Research Institute—University of Queensland, TRI Building, Woolloongabba, QLD 4102 Australia; 9 Queensland Brain Institute, University of Queensland, Brisbane, QLD 4072 Australia; 10 Department of Applied Sciences, Faculty of Health and Life Sciences, Northumbria University, Newcastle, Tyne and Wear NE1 8ST UK

**Keywords:** Cancer, Cell biology

## Abstract

**Background:**

Molecular characterisation of hepatocellular carcinoma (HCC) is central to the development of novel therapeutic strategies for the disease. We have previously demonstrated mutagenic consequences of Long-Interspersed Nuclear Element-1 (LINE1s/L1) retrotransposition. However, the role of L1 in HCC, besides somatic mutagenesis, is not well understood.

**Methods:**

We analysed L1 expression in the TCGA-HCC RNAseq dataset (*n* = 372) and explored potential relationships between L1 expression and clinical features. The findings were confirmed by immunohistochemical (IHC) analysis of an independent human HCC cohort (*n* = 48) and functional mechanisms explored using in vitro and in vivo model systems.

**Results:**

We observed positive associations between L1 and activated TGFβ-signalling, *TP53* mutation, alpha-fetoprotein and tumour invasion. IHC confirmed a positive association between pSMAD3, a surrogate for TGFβ-signalling status, and L1 ORF1p (*P* < 0.0001, *n* = 32). Experimental modulation of L1 ORF1p levels revealed an influence of L1 ORF1p on key hepatocarcinogenesis-related pathways. Reduction in cell migration and invasive capacity was observed upon L1 ORF1 knockdown, both in vitro and in vivo. In particular, L1 ORF1p increased PIN1 cytoplasmic localisation. Blocking PIN1 activity abrogated L1 ORF1p-induced NF-κB-mediated inflammatory response genes while further activated TGFβ-signalling confirming differential alteration of PIN1 activity in cellular compartments by L1 ORF1p.

**Discussion:**

Our data demonstrate a causal link between L1 ORF1p and key oncogenic pathways mediated by PIN1, presenting a novel therapeutic avenue.

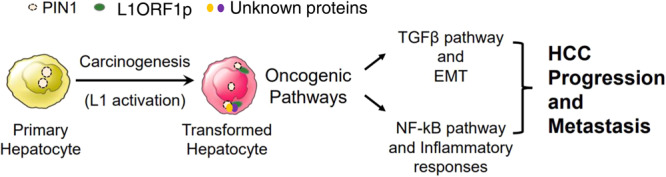

## Background

Hepatocellular carcinoma (HCC) is the sixth most common type of cancer and the fourth most frequent cause of cancer-related death worldwide [1]. HCC develops on the background of a chronic liver disease that has usually developed to the point of cirrhosis. In the context of an ongoing liver injury and/or metabolic disturbance, effectively three diseases are to be considered rather than cancer alone, making it a very difficult cancer to treat. Moreover, HCC is very heterogeneous at the pathological and molecular levels. To identify key drivers for targeted therapies, effort is now focused on understanding and defining molecular subclasses of HCC. To this end, Hoshida et al. used integrative transcriptome analysis and reported three clinically relevant subclasses of HCC S1-S3; S1 with aberrant activation of the WNT signalling pathway, S2 was characterised by proliferation as well as MYC and AKT activation, and S3 was associated with hepatocyte differentiation [2]. While histological subgroups related to gene mutations and molecular characteristics suggest the occurrence of 6 HCC classes (G1-G6) [3]. A more recent integration of genomic and transcriptomic HCC datasets from five data platforms by The Cancer Genome Atlas (TCGA) has supported three major clusters for HCC called as iClusters [4]. iCluster1 tumours exhibited features such as higher tumour grade and presence of macrovascular invasion, a low frequency of *CDKN2A* silencing and *CTNNB1* gene and *TERT* promoter mutations compared to iCuster2 and iCluster3. Overall, there was overexpression of proliferation marker genes and specific miRNA changes such as high expression of miR-181a and silencing of miR-122. In contrast, iCluster2 was associated with low-grade tumour and less microvascular invasion and exhibited enrichment for *HNF1A* mutation. iCluster3 was characterised by a higher degree of chromosomal instability with distinct 17p loss, high frequency of *TP53* mutation and hypomethylation of multiple CpG sites. Notably, an immune subclass of HCC has also been defined by transcriptional profiling alongside immunohistochemical examination, potentially identifying patients responding to immune checkpoint inhibitors such as nivolumab [5]. Based on this, 25% of HCC exhibited markers of inflammatory response such as high expression of PD-1, PD-L1 and active interferon-gamma signalling. This group was defined as “Immune class”. It contained two subtypes—“active immune” having markers of an adaptive T-cell response while “exhausted immune” exhibited activated stroma and infiltration of M2 macrophages. Another study specifically focused on the TGFβ pathway to classify HCC cases [6]. Approximately 40% of HCCs from the TCGA and the Catalogue of Somatic Mutations in Cancer datasets contain at least one somatic mutation in genes involved in the TGFβ signalling pathway, leading to dysregulated expression of genes such as *TGFB1*, *TGFB2, TGFBR1, SMAD3*, *SMAD4* and *SPTBN1*. Further transcriptomic analysis revealed, “activated”, “normal” and “inactivated” TGFβ signalling clusters in HCC cases, with patients in the inactivated cluster having poorest overall survival. However, non-disruption of the TGFβ pathway (normal) appeared to be associated with better outcome than either activation or inactivation of the pathway, underscoring the importance of the balance of this pathway in hepatocarcinogenesis.

Our previous work has demonstrated the activation and mutagenic consequences of long interspersed nuclear element (LINE-1, or L1) retrotransposition in HCC [7, 8]. L1 belongs to the “retrotransposon” category of mobile elements, as they utilise a “copy and paste” mechanism to jump to different genomic locations [9]. These elements are kept transcriptionally silent by epigenetic mechanisms, especially by methylation of a CpG island in the L1 promoter [7, 8, 10–14]. Global hypomethylation is a common feature of several cancers, including HCC, which can lead to L1 activation and expression of its proteins—L1 ORF1p and L1 ORF2p [15, 16]. Mobile L1 retrotransposons can drive insertional mutagenesis, causing altered gene structure and function. The process of L1 dysregulation and subsequent insertional mutagenesis has been recognised not only in HCC, but as a reported feature of multiple different epithelial cancers [17–20], however, a wider role of L1 mRNA and protein expression in cancer biology beyond retrotransposition has been suggested but is ill-defined. Aberrant L1 transcription may upregulate an interferon response associated with autoimmunity and age-associated inflammation [21–23]. L1 insertion can also lead to chimeric transcripts with adjacent genes, for example, L1-MET involving a fusion with the *c-MET* gene has been reported [24]. Likewise, L1-encoded proteins may interact with several host proteins and thus influence various signalling pathways [25]. The influence of L1 proteins on the DNA damage response has been described in several studies [26–28]. L1 ORF1p has also been implicated in inducing *hTERT* [29] and drug resistance [30, 31] in tumours, as well as a role in cancer progression via epithelial mesenchymal transition (EMT) [32].

In gastrointestinal (GI) cancer patients, L1 retrotransposition and expression correlated with clinical factors, including age and survival [33]. In addition, an inverse correlation between L1 retrotransposition and the expression of immune regulatory genes was observed i.e., tumours in the so-called high immune subgroup had significantly lower levels of L1 retrotransposition [33]. Also, the increased L1 expression has been associated with *TP53* gene mutation [16, 17]. This indicates that L1 expression in GI tumours is not random and may be related to a specific molecular subclass. Here, we analyse data from The Cancer Genome Atlas (TCGA) liver hepatocellular carcinoma (LIHC) study and combine the results with functional studies to dissect the influence of L1 expression in HCC. We reveal the distribution of L1 activation in the various HCC subclasses and identify L1 ORF1p as a novel activator of the TGFβ signalling and NF-κB-mediated inflammatory response pathways in human HCC and that this activation is largely mediated by PIN1.

## Materials and methods

### Patient samples and immunohistochemistry (IHC)

Archived diagnostic formalin-fixed paraffin-embedded (FFPE) biopsies with pathologically confirmed HCC were obtained from our own biobank, including patients diagnosed between 2002 and 2018, who consented to the use of their tissues surplus to diagnostic requirements for research purposes (project ID: 116370). Ethical approval was obtained for the use of FFPE HCC patient biopsies by the National Research Ethics Service (NRES) Committee North East (12/NE/0395). Clinical features were obtained from patient medical records. L1 ORF1p, pSMAD3 and PIN1 IHC was performed on a Ventana Discovery XT system. Details of antibodies are provided in Supplementary Table S4. An expert hepatic pathologist assessed and scored the staining based on intensity and number of positive cells. The pathologist was blind to clinical data.

### Cell lines and treatments

All cell lines (HepG2, Hep3B, PLC/PRF-5, HUH1, HUH7, SK-Hep1 SNU182 and SNU475) were cultured in RPMI1640 medium supplemented with 10% foetal bovine serum, l-glutamine and penicillin–streptomycin and were incubated at 37 °C with 5% CO_2_. All the cell lines were authenticated by serial tandem repeat (STR) profiling (NewGene, Newcastle, UK) and tested to confirm the lack of mycoplasma infection routinely.

For L1 ORF1 knockdown, Huh7 cells were transduced with lentiviruses encoding L1 ORF1-targeting (CCGGAAATGAAGCGAGAAGGGAAGTCTCGAGACTTCCCTTCTCGCTTCATTTTTTTTG) or control (non-targeting) shRNA (CCGGCAACAAGATGAAGAGCACCAACTCGAGTTGGTGCTCTTCATCTTGTTGTTTTTG) followed by puromycin selection (2 µg/ml, Sigma, P8833) as described previously [34].

For conditional overexpression, L1 ORF1 was cloned under control of a DOX-ON (doxycycline-inducible) promoter in a piggybac vector containing a puromycin resistance cassette. Hep3B cells were co-transfected with PB-DOX-empty or PB-DOX-L1 ORF1 along with plasmid expressing Transposase in order to integrate the piggybac vector using Trans-LT1 transfection reagent (Mirus Bio, 6003) as per the manufacturer’s instructions. Cells with stable integration were selected by puromycin selection (1 µg/ml). The cells were then induced with doxycycline (1 µg/ml, Sigma, D9891) for further experimentation.

For chemical treatment experiments, cells were seeded into 12- or 6-well plates, and 24 h after seeding cells were treated with the indicated agents (TGFβ (R&D Systems, 240-B-002), SB525334 (Selleckchem, S1476), DTM (Calbiochem, 5.30618.0001), BI605906 (Tocris, 5300)) at indicated doses and time.

### Immunocytochemistry (ICC)

Cells were cultured into 12-well plates containing glass coverslips and fixed with 4% formaldehyde (10 min) for staining using standard ICC protocols. Details of antibodies are provided in Supplementary Table S4. Finally, cells were mounted using ProLong Diamond Antifade Mountant (Invitrogen, P36961). Images were captured using 40 × /1.15NA APO oil lens on a Leica TCS SPE confocal microscope and analysed with LAS X (v 3.7.4) software for intensity measurements and Huygens pro 20.4 (www.svi.nl) software for co-localisation analysis [35].

### The IncuCyte® scratch wound assay

In total, 30,000 cells were seeded into 96-well plates to obtain confluence (at least five wells per cell line). Twenty-four hours later, a scratch wound was generated using an IncuCyte Wound Maker tool and plates were incubated and monitored in an IncuCyte time-lapse image capture system to visualise the initial wound and incremental wound closure at 6-h time intervals. Cell migration was then evaluated using an IncuCyte algorithm to measure wound confluence.

### Invasion assay

Invasion assay was performed using QCM ECMatrix cell invasion assay kit (Merck, ECM550). In short, 300 µl cell suspension (10^6^ cells/ml) was added at the top of the chamber in serum-free media. On the bottom, 500 µl complete media was added and the chambers were incubated at 37 °C and 5% CO_2_ for 72 h. Invaded cells were stained according to the manufacturer’s protocol. Phase-contrast images were taken (5–10 random fields) and quantified using ImageJ.

### Western blotting

Western immunoblotting of whole cell lysates was performed, as described previously [36]. Details of primary antibodies are provided in Supplementary Table S4. Images represent one of the three independent repeats and quantification is done using ImageJ.

### Luciferase assay

PAI1 promoter luciferase reporter to monitor SMAD3 activity and Renilla constructs were co-transfected in a ratio of 10:1 using Trans-LT1 transfection reagent and the enzyme activities were determined using the Dual-Luciferase reporter assay system (Promega, E1910) and Omega FLUOstar plate reader (BMG Labtech Ltd., Aylesbury, UK) with luminescence settings.

### RNA extraction and quantitative real-time polymerase chain reaction (qRT-PCR)

Total RNA was extracted using an RNeasy Mini Kit (Qiagen, 74104). The RNA purity and concentration were estimated with an ND-1000 spectrophotometer (NanoDrop Technologies, Thermo Fisher Scientific). Complementary DNA was generated using the cDNA Reverse Transcription Kit (Promega, A3500) as per the manufacturer’s instructions. The qRT-PCR was carried out as described previously [36]. Primers used in the study are listed in Supplementary Table S5.

### Cell lines RNAseq analysis

Total RNA was isolated for all the indicated cell lines from three biological replicates using an RNeasy kit. RNA integrity was confirmed by Agilent Bioanalyser (RIN >9.5 for all the samples). Illumina Tru-seq paired-end strand-specific sequencing was carried out on a NextSeq500 (Newcastle University Genomics Facility). Post-trimming quality control was performed with FastQC (version: 1.0.0). The resulting FastQ files were mapped on to human reference genome using RNAseq alignment tool (V1.1.1 for Huh7 and V2.0.2 for Hep3B) using Illumina BaseSpace software. A differential expression gene (DEG) list was obtained using DESeq2. Gene set enrichment analysis (GSEA) was carried out using broad institute’s GSEA software (V4.0.3), wherein “hallmark gene sets” from a molecular signatures database (MSigDB) were analysed by contrasting full gene lists of Huh7-L1KD versus Huh7-NT and Hep3B-DOX-Empty versus Hep3B-DOX-L1 ORF1 samples. Upstream regulators and interaction networks of DEGs were analysed using Qiagen Ingenuity Pathway Analysis (IPA, Winter Release, Dec 2021) software. The data can be found in GEO database (GSE126615 and GSE194251).

### L1 transcript analysis

Human HCC RNAseq data was downloaded from the TCGA-LIHC project and mapped to the human L1-Ta sequence (5’UTR-promoter, Genbank: L19092) by BLAT alignment using an in-house algorithm to obtain L1 counts [37]. The counts were normalised by the total number of reads in each library and expressed here as counts per million. We specifically focused on L1-Ta because these are the most recently integrated L1 elements within the human genome belonging to the human-specific L1 (L1Hs) family.

### Mice and in vivo experimentation

All animal experiments were approved by the Newcastle University Ethical Review Committee and performed under a UK Home Office licence in accordance with the ARRIVE guidelines (http://www.nc3rs.org.uk/page.asp?id=1357). Experiments were performed using 8–10-week-old male NSG mice obtained from Charles River. Mice under isoflurane general anaesthesia underwent laparotomy to expose the liver. Using an insulin syringe, 1 million cells (labelled with zsGreen-luciferase construct using a lentiviral vector) in a volume of 30 µl of 50% matrigel were injected directly into the left lobe of the liver. Whole-body bioluminescence images were acquired using an In Vivo Imaging System (IVIS spectrum: Perkin Elmer). Prior to in vivo imaging (10 min), mice were injected intraperitoneally with 10 mg/Kg of d-luciferin (Perkin Elmer). Following in vivo monitoring of tumour development, mice were humanly euthanised, organs were harvested and ex vivo imaging was performed. Both in vivo and ex vivo images were acquired using an open filter with auto-exposure. Images were analysed using Living Image (version 4.7.2, Perkin Elmer). In vivo and ex vivo photon signals were quantified using the region of Interest method of analysis [38]. Harvested organs were fixed in absolute ethanol and then embedded in paraffin for IHC analysis (Supplementary Methods for details).

### Statistical analysis

L1 transcript expression distribution amongst HCC subgroups (predefined clusters—information obtained from previous publications) and patients’ clinical features was analysed using the SPSS statistical package (IBM, version 25). Likewise, IHC data, along with patients’ clinical features, were analysed by the SPSS statistical package. Categorical and continuous datasets were compared using Pearson’s chi-square (Fishers Exact for groups with <5 cases) and Wilcoxson signed ranks (two groups, paired data) or Mann–Whitney (two groups) or Kruskal–Wallis (three groups) tests, respectively. Correlations between normalised L1 counts and a subset of genes were calculated by the Spearman correlation test. GraphPad Prism software (GraphPad 8.0) was used for cellular assays and analysed by Student’s *t* test (two groups) or one- or two-way ANOVA with Tukey’s multiple comparison when required (three groups). Fold change (FC) data were analysed by one-sample *t* test using 1 as the hypothetical mean. *P* < 0.05 was taken as a cut-off for significance. Mean ±  standard errors or median lines are shown in figures where applicable. *P < 0.05, **P < 0.01, ***P < 0.001, ****P < 0.0001.

## Results

### Analysis of TCGA-HCC cohort revealed positive associations between L1 transcripts and *TP53* mutation, the TGFβ signalling pathway, tumour invasion and AFP levels

**Fig. 1:**
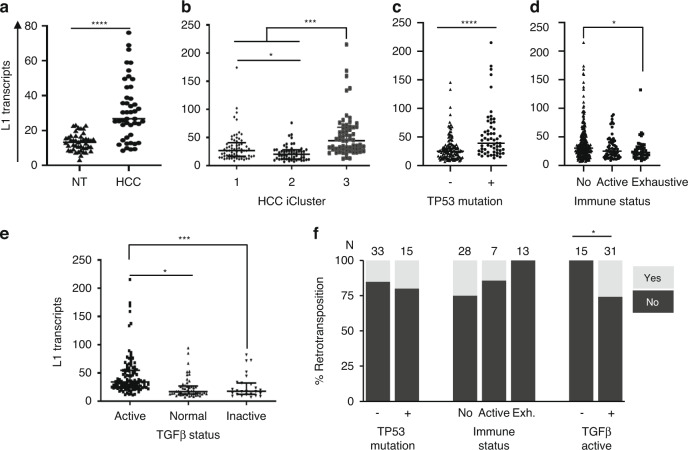
Characterisation of L1 expression and retrotransposition in human HCC. **a** Graph represents L1 transcript normalised counts of total RNAseq of matched HCC and non-tumour (NT) tissues, *n* = 45, ****P < 0.0001, Wilcoxson text. **b**–**e** Graphs representing L1 transcripts distribution amongst HCC cases classified as iClusters, *n* = 182, *P < 0.05, ***P < 0.001, Kruskal–Wallis test with multiple comparison (**b**), *TP53* mutation status, *n* = 187, ****P < 0.0001, Mann–Whitney test (**c**), immune status (no = non-immune, active and exhaustive), *n* = 372, *P < 0.05, Kruskal-Wallis test with multiple comparison (**d**) and TGFβ status, *n* = 138, *P < 0.05, ***P < 0.001, Kruskal–Wallis test with multiple comparison (**e**); **f** Graph representing prevalence of somatic retrotransposition activity amongst HCC cases classed as *TP53* mutation, immune status (no = non-immune, active and Exh. = exhaustive) or active TGFβ signalling status. The numbers on top represent the number of cases in each group. *P < 0.05, Fisher’s exact test.

RNAseq data (TCGA-HCC samples) revealed a significant increase in L1 transcript levels in HCC compared to matched non-tumour (NT) tissues (median 26.73 versus 16.25, respectively, Fig. 1a). In terms of HCC subclasses, L1 expression was highest in iCluster3 (characterised by a higher degree of chromosomal instability, high frequency of *TP53* mutation, and hypomethylation of multiple CpG sites [4]) (Fig. 1b). As expected, L1 expression associated positively with *TP53* mutation (median 38.97 vs 25.08 for *TP53* mutant versus wild type, (Fig. 1c), with a negative correlation between L1 transcripts and TP53 target gene signature calculated based on 20 transcripts belonging to TP53 target genes [4] (Spearman *r* = −0.272, *P* = 0.0002). Besides *TP53*, no significant associations were observed with any other common HCC gene mutations such as *CTNNB1* or *TERT* promoter. Similar to GI cancers [33], L1 expression negatively correlated with immune-rich HCC. In particular, L1 expression was significantly lower in HCC belonging to the exhaustive immune subclass (median 22.79 versus 25.18 and 30.35 in exhaustive versus active immune and non-immune HCC, respectively, Fig. 1d). Further examination of HCC subclasses based on their TGFβ signalling pathway status, as defined by an 18-gene TGFβ superfamily gene signature [6], showed L1 expression to be significantly higher in samples with an activated TGFβ pathway when compared to normal or inactivated signalling groups (Fig. 1e). In addition, 14 of the 18 members of the TGFβ signalling superfamily showed significant correlation of expression with that of L1 expression (Supplementary Fig. S1). To explore these associations with other L1 subfamilies (L1Hs, L1PA2, L1PA3 and L1BPa1) we looked into published L1 counts for a subset of TCGA-HCC samples [39]. L1 counts obtained by our analysis correlated significantly with the published counts (*P* < 0.001, Supplementary Fig. S2). We have also confirmed significant L1 associations as we have previously observed in our whole dataset, with L1 subfamilies, in the subset samples. In the subset, significant associations between L1-Ta and iCluster3, *TP53* mutation and TGFβ activation, as they were for other subfamilies, with the exception of L1PA3 and *TP53* mutation (data not shown).

Recently, the pan-cancer analysis of the whole genome (PCAWG) consortium reported active retrotransposition in various cancer types, including a subset of TCGA-HCC samples [17]. We combined the information to evaluate the molecular associations of somatic L1 retrotransposition in HCC. Although not statistically significant, we observed trends towards a positive association with *TP53* mutations and a negative association with immune cell-enriched cancers, as reported for other cancer types. In contrast, we did identify a significant positive association between somatic L1 retrotransposition and active TGFβ signalling pathway, which has not been previously reported for any other cancer type (Fig. 1f).

To analyse the relationship between L1 expression in HCC and other clinical characteristics of the patients in the TCGA cohort, we parsed the dataset with clinical characteristics including age, gender, ancestry, aetiology, pathological grade, patient AFP levels and tumour invasion. For this analysis, HCC samples were divided into two groups based on their L1 transcript expression level—L1-Low (L1 expression equivalent to non-tumour (NT) liver tissue, L1 norm count ≤25) and L1-High (L1 expression higher than NT liver tissue). There were no significant correlations with age, gender, ancestry or aetiology (Supplementary Table S1). However, L1-High expression was significantly associated with elevated serum AFP levels, with more advanced TNM stage and vascular invasion (Supplementary Table S1). L1-high expression was also significantly associated with poorer histological tumour grade, intratumoral fibrosis and cholestasis. Thus, high L1 expression was associated with dedifferentiated invasive tumours (Supplementary Table S1).

### Independent L1 ORF1p immunohistochemical (IHC) analysis confirmed an association with TGFβ signalling

**Fig. 2:**
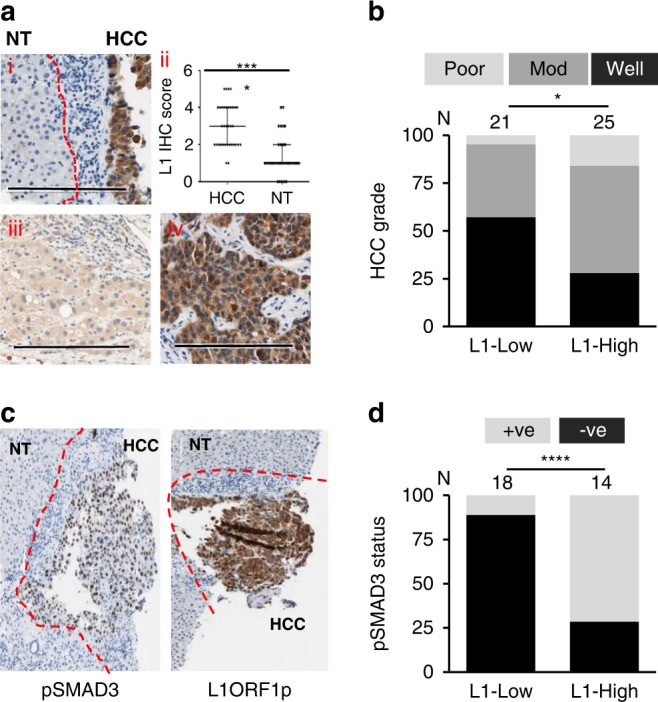
Immunohistochemical (IHC) analysis of L1 ORF1p in HCC and its association with clinical features. **a** Representative images of L1 ORF1p IHC in HCC diagnostic biopsies (scale bar = 200 µm) (i) represents L1-positive HCC tissue with adjacent non-tumour (NT) tissue and immune infiltrates having minimal positivity; red dashed line marks the margin of HCC tissue along with adjoining immune infiltrate. ii represents L1 ORF1p IHC scores for HCC and NT surrounding tissues, *n* = 34. iii–iv represent HCC cases belonging to L1-Low and L1-High class, respectively. **b** Graph represents change in prevalence of tumour grade (poor, moderately (mod) or well-differentiated) depending upon HCC L1 ORF1p status, *P < 0.05 chi-square test between tumours well-differentiated or not. Numbers on top represents cases in each group. **c** Representative image of pSMAD3 IHC in HCC and L1 ORF1p IHC of the same sample; red dashed line marks the margin of HCC tissue and adjoining immune infiltrate. **d** Graph representing prevalence of pSMAD3 positivity in HCC depending upon HCC L1 ORF1p status. Numbers on top represents cases in each group. +ve = pSMAD3 positive nuclei present; −ve = absence of pSMAD3 positive nuclei. ****P < 0.0001, Fisher’s exact test.

Transcript expression analysis is limited in terms of its ability to distinguish authentic full-length transcripts and non-functional L1 RNAs, while RNAseq data also has limitations due to variability of tumour purity of the samples. We therefore validated the observations made above by evaluating the levels of L1 ORF1 protein (L1 ORF1p) expression by IHC on formalin-fixed paraffin-embedded (FFPE) diagnostic biopsies from HCC patients in an independent cohort from our own biobank. The results were examined in relation to tumour grade, clinical features and patient outcome. Similar to the transcript data, L1 ORF1p expression was observed in HCC with minimal background expression in surrounding NT tissues or immune infiltrates (Fig. 2ai–ii). The L1 ORF1p IHC results were analysed and quantified by a pathologist (blinded for clinical data) based on intensity level and percentage positivity. L1 ORF1p was mostly cytoplasmic and all tumours stained positively for L1 ORF1p expression. Although the intensity of staining varied between patient tumour samples, individual biopsies showing mostly a uniform distribution of L1 ORF1p expression throughout the tumour tissue. The HCC samples were categorised into 2 groups—L1-Low and L1-High—for further analysis (Fig. 2aiii–iv). Despite the smaller numbers of cases, the level of L1 expression (classed as L1-High or L1-Low) was associated with tumour grade, where L1-High was more common in poorly differentiated tumours (*P* = 0.046, chi-square test) (Fig. 2b and Supplementary Table S2). There were no significant correlation between HCC L1 status and TNM stage in our cohort, however, the number of cases are too low in certain categories and thus this needs to be verified in a larger cohort. In addition, there was no significant difference in Kaplan–Meier survival analysis comparing L1-Low and L1-High categories in the whole cohort; however, focusing on the patients that had undergone any kind of treatment for HCC, a trend towards association between L1 expression and poorer patient outcome was observed and this was especially significant in the patients undergoing transarterial chemoembolisation (TACE) therapy. Although significant, the number of cases were quite low (*n* = 24) and data needs to be validated in a larger cohort (Supplementary Table S2).

Finally, we carried out IHC for phosphorylated SMAD family member 3 (pSMAD3-ser425) as a surrogate for TGFβ signalling activation status in the tumours in a subset of the initial L1-IHC cohort (Fig. 2c). pSMAD3 showed nuclear positivity and HCC samples varied in percentage positivity for pSMAD3 from totally absent to up to >90% positive. A significant association was observed between pSMAD3 positivity and L1 ORF1p status of HCC (Fig. 2d). Thus, the data from IHC analysis complemented the transcriptional analysis data and supported the positive association between L1 activation and activated TGFβ signalling.

### Targeting L1 ORF1 led to the downregulation of tumorigenesis and tumour invasion

Western blotting was performed on whole cell lysates of various liver cancer cell lines to evaluate the expression status of L1 ORF1p and to select cell lines for in vitro L1 manipulations. A range of L1 ORF1p expression was observed in these cell lines, as seen previously in primary HCC samples. L1 ORF1p expression was high in SNU475, SNU182 (mesenchymal-like cell lines), SK-Hep1 (cholangiocarcinoma) and Huh7 (epithelial cell line), and lower in the other epithelial cell lines Huh1, PLC/PRF-5 and Hep3B while being almost undetectable in HepG2 (epithelial cell line) (Supplementary Fig. S3A). L1 ORF1p expression was also confirmed in six out of eight of the cell lines by FACS analysis and the results corroborated with the western blotting data (Supplementary Fig. S3B). Coulouarn et al. performed a transcriptomic analysis of various liver cancer cell lines and classed them as having an early- or late-TGFβ response signature [40]. In addition, Coulouarn et al. also demonstrated that cell lines having a late-TGFβ response signature displayed higher migration and invasion capacities when compared to cell lines having an early-TGFβ response [40]. All three of the cell lines belonging to the category reported to have a late-TGFβ response have readily detectable L1 ORF1p expression (Supplementary Fig. S3A). Likewise, in a recent study, genetic, RNA, and protein profiles of liver cancer cell lines were compared and overall the cell lines were grouped into three major classes CL1 (hepatoblast-like), CL2 (mixed epithelial mesenchymal) and CL3 (mesenchymal-like) [41]. Again, amongst the cell lines analysed here, L1 ORF1p expression was higher in cell lines belonging to CL3 group (Supplementary Fig. S3A). Cell lines belonging to the early-response or CL1 group were mixed in terms of L1 ORF1p expression (Supplementary Fig. S3A).

**Fig. 3:**
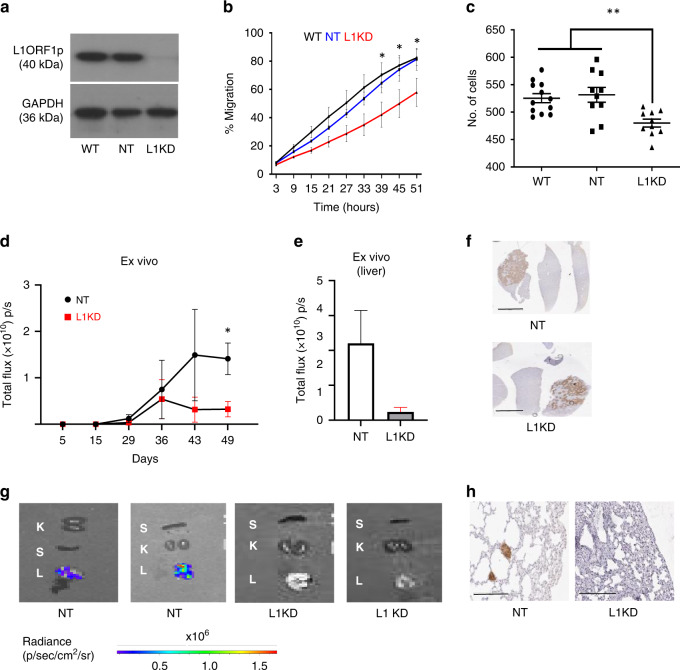
Influence of L1 knockdown on Huh7 cells. **a** Western blot confirming knockdown of L1 ORF1p in L1KD cells expressing L1-specific shRNA versus cells expressing non-targeting shRNA (NT) and wild-type (WT) cells. GAPDH is used as a loading control. **b** Graph representing cell migration measured by the closure of scratch wound using automated incucyte system. Values represent combination of three independent experiments. *P ≤ 0.05 for L1KD versus NT and WT from 39 h onwards, two-way ANOVA. **c** Graph representing number of cells invaded through Boyden chamber in 72 h. Data show combination of 3 independent repeats. **P < 0.01, one-way ANOVA with multiple comparisons. **d** Graph represents tumour growth upon injection of luciferase labelled Huh7-NT and Huh7-L1KD cells when injected intrahepatically in NSG mice (*n* = 4) per group, as monitored by IVIS imaging. Sample number was decided based on our previous experience of 100% engraftment. Mice were randomly selected to put in the two groups. *P < 0.05, unpaired *t* test. **e** Graph represents ex vivo liver signal in the two groups. **f** Representative images of tumours developed in the liver visualised by IHC using anti-luciferase antibody. Background liver showing minimal positivity. Scale bar = 4 mm. **g** Ex vivo images of harvested organs (L   lung, K   kidney, S   spleen) in the indicated groups as visualised by IVIS imager. **h** Luciferase IHC in the lung of indicated groups to identify metastatic cells.

To test the effect of knocking down L1 ORF1 in a cancer cell that expresses L1 ORF1p and whether it influenced tumour biology, shRNA-based stable knockdown of L1 ORF1 in Huh7 cells was performed. Western blotting and FACS analysis confirmed downregulation of L1 ORF1p to undetectable levels in Huh7-L1KD cells, in comparison to wild-type (Huh7-WT) and control cells expressing non-targeted shRNA (Huh7-NT) (Fig. 3a and Supplementary Fig. S4A). A significant reduction in cell migration (Fig. 3b) and cell invasive capacity (Fig. 3c) was observed upon L1 ORF1 knockdown. Moreover, we observed reduced in vivo tumorigenecity for Huh7-L1KD cells compared to Huh7-NT when injected intrahepatically in NSG mice (cell lines were labelled using lentiviral particles containing zsGreen-luciferase construct for in vivo imaging), assessed using IVIS imaging (Fig. 3d, e), although there was no difference in growth rate of Huh7 cells upon L1 knockdown in vitro (Supplementary Fig. S4B). Expression of luciferase in the tumours of both the groups was also confirmed by IHC of liver sections using anti-luciferase antibody (Fig. 3f and Supplementary Fig. S4C). Overall, tumours formed by the Huh7-NT group were larger, with more necrotic areas compared to the Huh7-L1KD group (Supplementary Fig. S4D). The edges of these necrotic areas were positive for pSMAD3 (Supplementary Fig. S4E). Moreover, Huh7 cells were present in the lungs of three out of four mice in the Huh7-NT group. In all, 2/3 were positive for luciferase signal in the lung on ex vivo imaging, while in 1 mouse was humanely killed earlier, having reached clinical endpoints, positivity was verified by IHC using an anti-luciferase antibody (Fig. 3g, h). These data indicated an increased metastatic migration from liver to lung when L1 was expressed. No luciferase signal was observed in the lungs or any other organ except liver, in the Huh7-L1KD group (*n* = 4) (Fig. 3g, h).

**Fig. 4:**
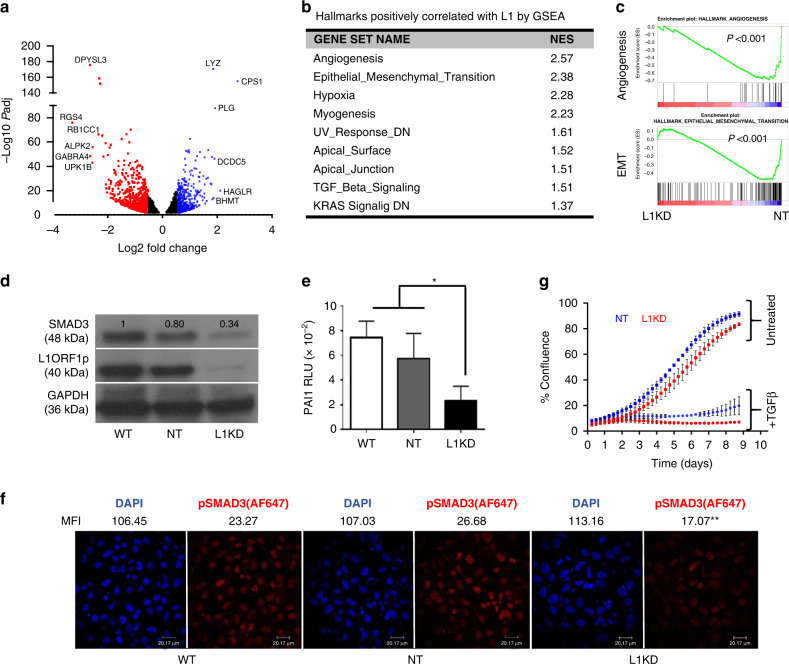
Influence of L1 knockdown on signalling pathways in Huh7 cells. **a** Volcano plot representing differentially expressed genes (*P*adj < 0.05) upon RNAseq analysis of Huh7-L1KD versus Huh7-NT cells. Fold change (FC) and *P*adj values were obtained by DEseq2 analysis of RNAseq data. Blue dots represent upregulated (log2FC > 0.5) and red dots represent downregulated genes (log2FC < −0.5). **b** Table showing hallmark pathways found significantly enriched (FDR < 25%, *P* < 0.05) in Huh7-L1KD cells compared to Huh7-NT cells by GSEA (gene set enrichment analysis), NES   Normalised Enrichment Score. **c** Enrichment plots generated by GSEA. **d** Western blot analysis of indicated Huh7 transgenic cells showing levels of indicated proteins. GAPDH was used as a loading control. The numbers on top of each band represent fold change expression with respect to WT cells normalised using GAPDH. **e** *PAI1*-luciferase assay in indicated cell lines, bars represent relative luciferase units (RLU) normalised by Renilla. Data are the combination of three independent biological experiments done in triplicates. *P < 0.05, one-way ANOVA with multiple comparisons. **f** Immunocytochemical images showing pSMAD3 staining in Huh7 wild-type (WT), non-targeted shRNA control cells (NT) and L1 knockdown cells (L1KD). Cells were stained with mouse-anti-pSMAD3 antibody followed by anti-mouse-AF647 secondary antibody. DAPI was used to counterstain the nuclei. MFI   mean fluorescent intensity of the indicated channel (*n* = 32–35). **P < 0.01 for L1KD versus NT and WT for AF647 channel, one-way ANOVA with multiple comparisons. **g** Growth curves of indicated cells with and without TGFβ treatment as measured by incucyte system. The graph represents combination of two independent experiments, each done in quadruplets.

RNAseq analysis of Huh7-L1KD versus Huh7-NT cells revealed 1512 differentially expressed genes (DEGs) when a cut-off of log2 fold change 0.5, *P*adj <0.05 was used, while 334 DEGs with a cut-off of log2 fold change 1, *P*adj < 0.05 (Fig. 4a). Gene set enrichment analysis (GSEA) using expression data of the complete gene list revealed 9/50 hallmark gene sets (MSigDB collection), including angiogenesis, EMT and TGFβ signalling positively correlating with higher L1 ORF1 expression with FDR < 25% and *P* < 0.05 (Fig. 4b, c and Supplementary Table S3A). In addition, the *SMAD3* transcript was differentially expressed, not *SMAD4* or *SMAD2*, in Huh7-L1KD versus NT cells (log2FC −0.72, *P*adj = 1.40E-15 for *SMAD3* while log2FC −0.23, *P*adj = 0.009 for *SMAD4* and log2FC −0.23, *P*adj = 0.03 for *SMAD2* by DEseq2 analysis of Huh7-NT_vs_Huh7-L1KD). Moreover, Huh7-L1KD cells exhibited downregulation of SMAD3 at the protein level, when compared to Huh7-NT cells (Fig. 4d). The data support our conclusion that L1 ORF1p upregulates TGFβ signalling. We subsequently confirmed the downregulation of TGFβ signalling in Huh7-L1KD cells using the *PAI1*-promoter luciferase reporter assay, which contains 3 CAGA boxes (SMAD3/SMAD4 binding site) [42] (Fig. 4e) and with pSMAD3 immunofluorescence staining (Fig. 4f). Of note, despite reduced basal-TGFβ signalling, the anti-proliferative response to a TGFβ stimulus was maintained in the Huh7-L1KD cells (Fig. 4g). We attributed this to *SMAD3* being a direct target of TGFβ signalling in these cells [43]. Supporting this, an increase in SMAD3 expression at both protein and transcript levels was observed in both Huh7-L1KD and NT cells upon TGFβ stimulation (Supplementary Fig. S5A, S5B).

Previously, TGFβ treatment has been shown to upregulate L1orf1p expression in BEAS-2B (human bronchial epithelial) cells showing the influence of TGFβ1 upstream of L1 [44]. However, no evident increase in L1orf1 protein or transcript was observed in Huh7 upon TGFβ treatment (Supplementary Fig. S5C, S5D). SMAD3 was used as a readout for the response to TGFβ treatment and was substantially upregulated upon TGFβ treatment in a dose-dependent manner (Supplementary Fig. S5C, S5D).

### L1 ORF1p upregulates cytoplasmic PIN1—a potential mechanism of influencing oncogenic pathways

To further explore the mechanism of influence of L1 on TGFβ signalling, we investigated common regulators/interacting partners of L1-encoded proteins (L1 ORF1p and L1 ORF2p) and TGFβ signalling. These included PIN1, which is a propyl isomerase that isomerises specific phosphorylated Ser/Thr-Pro proteins and is overexpressed in various cancer types [45]. PIN1 binds to phospho-L1 ORF1p, and phosphorylation of the interaction sites (S18 and S27) is essential for retrotransposition [46]. In addition, Pin1 has been reported to be induced in fibrotic liver and demonstrated to be essential for TGFβ1-mediated fibrogenic signalling [47]. PIN1 also binds to pSMAD3, inducing a conformational change and facilitating its degradation [48]. Thus, PIN1 can act as a negative regulator of SMAD3.

**Fig. 5:**
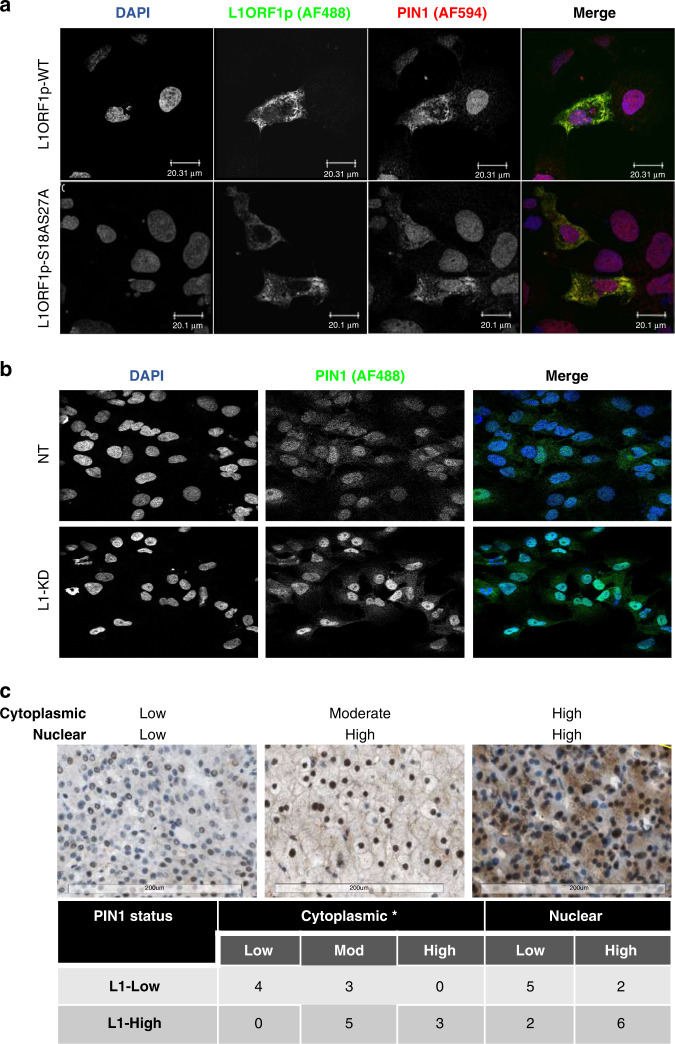
L1 ORF1p influences PIN1 cellular localisation. **a** Immunocytochemical (ICC) images of Hep3B cells transfected with plasmid expressing L1 ORF1p wild-type (WT) or S18AS27A mutant form and costained for PIN1 and L1 ORF1p. Anti-PIN1 antibody was directly conjugated with AF-594 and L1 ORF1p was visualised using anti-L1 ORF1p with an anti-mouse-AF488 secondary antibody. DAPI was used to counterstain nuclei. Scale bar = 20.1 µm. **b** ICC images showing Pin1 staining in Huh7 non-targeted shRNA control cells (NT) and L1 knockdown cells (L1KD). Cells were stained with mouse-anti-PIN1 antibody followed by anti-mouse-AF488 secondary antibody. DAPI was used to counterstain the nuclei. **c** Representative PIN1 IHC images (scale bar = 200 µm) showing PIN1 cytoplasmic and nuclear status on top. The table below represents number of cases belonging to indicated categories. *P < 0.05, chi-square test.

Co-staining for L1 ORF1p and PIN1 in Hep3B cells (cell line with low endogenous L1 ORF1p expression, Supplementary Fig. S3A) transiently transfected with a plasmid encoding L1 ORF1p confirmed co-localisation of PIN1 with L1 ORF1p in transfected cells. Notably though, increased expression of PIN1 in the L1 ORF1p transfected cells was observed in the cytoplasm (Fig. 5a and Supplementary Fig. S6A, S6B). Since PIN1 is a negative regulator of TGFβ signalling through its binding with pSMAD3 in the nucleus [48], L1 ORF1p may potentially upregulate TGFβ signalling by sequestering PIN1 in the cytoplasm. Redistribution of PIN1 was also observed upon L1 ORF1 knockdown in Huh7 cells; being more nuclear in Huh7-L1KD compared to Huh7-NT cells (Fig. 5b). In addition, a reduction in total PIN1 protein level was observed in Huh7-L1KD cells compared to Huh7-NT or WT cells (Supplementary Fig. S7).

Transfection with a mutant L1 ORF1p (L1 ORF1p-S18AS27A [46]), which is reported to not interact with PIN1, increased total and cytoplasmic expression of PIN1 similar to WT L1 ORF1p, with relatively less co-localisation (extent of spatial overlap between two fluorophores) (Fig. 5a and Supplementary Fig. S6B, S6C). We concluded that the direct interaction of L1 ORF1p with PIN1 was not essential for the upregulation of cytoplasmic PIN1.

In human HCC biopsies studied by IHC, PIN1 positivity was observed in 15 out of 17 HCC, as either nuclear or both nuclear and cytoplasmic expression. The relationship between L1 ORF1p and PIN1 intensity, percentage positivity and localisation was assessed and scored by a liver pathologist in a blinded manner (Fig. 5c). This confirmed that cytoplasmic PIN1 expression in situ, was significantly associated with L1 ORF1p expression. In a number of other cancer types, PIN1 overexpression and cytoplasmic localisation of PIN1 has been associated with cancer aggressiveness and metastasis [49–51]. While the L1 ORF1p motif essential for influencing PIN1 in HCC is as yet unknown, we explored the impact of cytoplasmic PIN1 further.

**Fig. 6:**
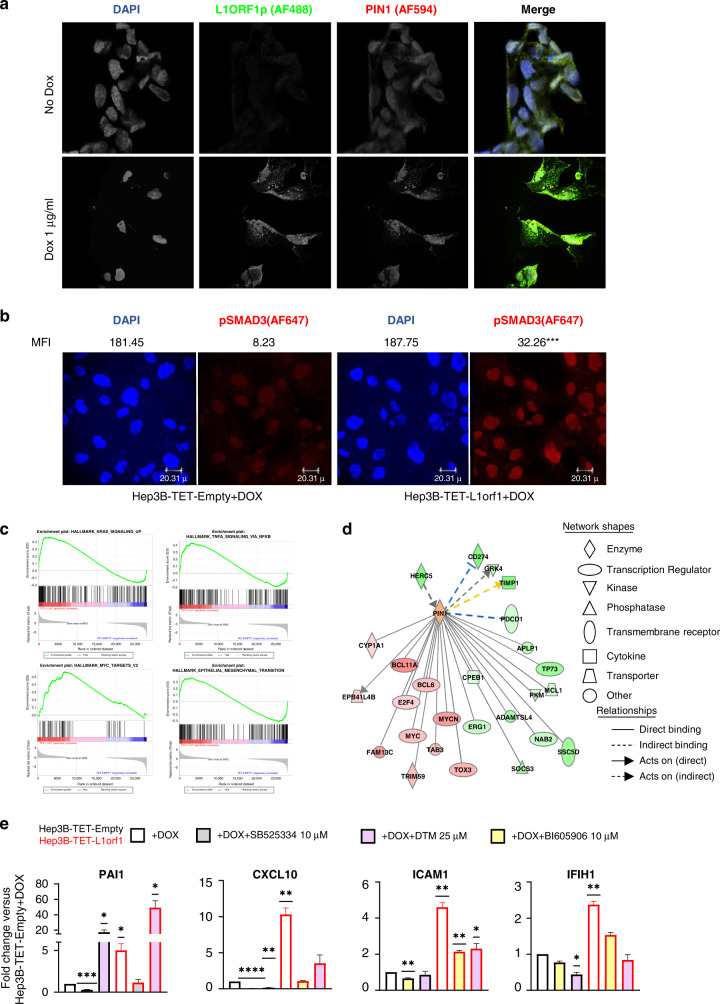
Influence of L1 ORF1p overexpression on Hep3B cell lines. **a** Immunocytochemical (ICC) images of Hep3B-DOX-L1 ORF1 cells costained with PIN1-AF-549 and L1 ORF1 + AF488 48 h after Dox induction. Nuclei were counterstained with DAPI. Untreated cells were used as controls. Co-localisation analysis for Pin1 and L1 ORF1p revealed Manders coefficient M1 (proportion of the intensity from the green channel that co-localises with the red channel) = 0.958 and M2 (proportion of the intensity from the red channel that co-localises with the green channel) = 0.97; Pearson’s coefficient (co-variance of the two channels) = 0.898 for the image. **b** ICC images showing pSMAD3 staining of Hep3B-DOX-Empty and Hep3B-DOX-L1 ORF1 cells 48 h after Dox induction. Cells were stained with mouse-anti-pSMAD3 antibody followed by anti-mouse-AF647 secondary antibody. DAPI was used to counterstain the nuclei. MFI = mean fluorescent intensity of the indicated channel (*n* = 20). ****P < 0.0001 for AF647 channel, unpaired *t* test. **c** Enrichment plots obtained by GSEA upon analysing RNAseq data comparing Hep3B-DOX-L1 ORF1 versus Hep3B-DOX-Empty cells. The significance cut-off was set at FDR < 25% and p < 0.05. **d** Interaction map of PIN1 with differentially expressed genes in Hep3B-DOX-L1 ORF1 versus Hep3B-DOX-Empty cells. Shades of green represent downregulated and red represents upregulated genes. The orange colour of PIN1 indicates IPA predicts activation of PIN1 activity. **e** Graphs representing RT-qPCR results of indicated transcripts in Hep3B-DOX-Empty and Hep3B-DOX-L1 ORF1 cells upon Dox induction (1 µg/ml for 48 h) alone or in combination with the indicated inhibitor for the last 24 h. 18S was used as a normalisation control. Values represent mean ± SE of three independent experiments. *P < 0.05, **P < 0.01, ***P < 0.001, ****P < 0.0001, one-sample *t* test with a theoretical mean of 1.

To evaluate the influence of L1 ORF1p on the cellular transcriptome, we generated a conditional (TET/DOX-ON) L1 ORF1 overexpression system using a piggybac vector in Hep3B cells (cell line with low endogenous L1 ORF1p expression, Supplementary Fig. S3A). Cells containing an empty piggybac vector were generated as controls (Supplementary Fig S9). In our system, Doxycycline (Dox) induction switched on L1 ORF1p expression, which was found in association with cytoplasmic aggregation of PIN1 (Fig. 6a). The cytoplasmic punctate pattern of PIN1 in Hep3B-DOX-L1 ORF1p + Dox cells was similar to the pattern of L1 ORF1p, with no aggregation of PIN1 observed in the absence of L1 ORF1p overexpression (Fig. 6a and Supplementary Fig. S8). In addition, basal pSMAD3 was upregulated in the Hep3B DOX-L1 ORF1p + Dox cells compared to Hep3B DOX-Empty + Dox cells (Fig. 6b).

Next, using the L1 ORF1p overexpression inducible system, we performed RNAseq analysis on Hep3B cell lines 72 h post Dox induction, evaluating the influence of L1 ORF1 overexpression on the overall transcriptome. In total, 1426 differentially expressed genes (DEGs) were found in Hep3B-DOX-L1 ORF1 cells when compared to Hep3B-DOX-empty cells at a cut-off of log2 Fold change 0.5, *P*adj < 0.05. GSEA using expression data of the complete gene list was performed, focusing on gene signatures associated with higher L1 ORF1 expression, using an FDR < 25% and *P* < 0.05. Analysis revealed 18/50 hallmark gene sets (MSigDB collection) correlating positively with L1 ORF1, including those for KRAS upregulated signalling, Myc targets, inflammatory responses (TNFalpha, interferon alpha and gamma) and EMT. We concluded that L1 ORF1 overexpression influenced these tumorigenesis-associated pathways (Fig. 6c and Supplementary Table S3B).

We went on to further interrogate the potential influence of PIN1 within these regulatory networks, using Ingenuity Pathway Analysis (IPA). An interaction network of PIN1 with the DEGs was generated, identifying 26 nodes and predicted PIN1 to be activated in Hep3B-DOX-L1 ORF1 compared to Hep3B-DOX-empty cells (Fig. 6d). Besides DEGs, PIN1 is known to influence the activity or expression of several upstream regulators of the DEGs, including TGFB1 and NF-κB complex (Supplementary Fig. S10A). Similarly, the interaction network of PIN1 with Huh7-L1KD versus Huh7-NT DEGs revealed 41 interaction nodes, with PIN1 predicted to be downregulated in Huh7-L1KD compared to Huh7-NT cells (Supplementary Fig. S10B). PIN1 is known to influence several upstream regulators of these DEGs as well (Supplementary Fig. S10C). These data support a central role for PIN1 in the regulatory network of the L1 ORF1p-induced inflammatory (NF-κB) response and TGFβ signalling.

RT-qPCR confirmed upregulation of *PAI1* (SMAD3 target upon TGFβ stimulation) and *CXCL10, IFIH1* and *ICAM1* (inflammatory or NF-κB response genes) in the Hep3B-DOX-L1 ORF1p + Dox cells. The L1 ORF1p-induced upregulation of *PAI1* was suppressed by treatment with SB525334, a specific inhibitor of the TGF-beta receptor I (ALK5) receptor. Upregulation of *CXCL10, IFIH1* and *ICAM1* was suppressed by treatment with BI605906, a specific inhibitor of IKKβ—a key activatory kinase of the NF-κB signalling pathway (Fig. 6e). These data confirmed the involvement of the respective pathways. Moreover, PIN1 inhibition using small molecule inhibitors DTM (binds to PIN1 active pocket [52]) and KPT-6566 (covalent PIN1 inhibitor [53]), abrogated the influence of L1 ORF1p on NF-κB response genes, while further activating TGFβ signalling (Fig. 6e and Supplementary Fig 10D). Thus, in these cells PIN1 activates NF-κB and inhibits TGFβ signalling. Since PIN1 inhibits p65 (a NF-κB subunit) binding to IκBα in the cytoplasm leading to enhanced p65 nuclear localisation, thus activating the NF-κB pathway [54] and downregulates TGFβ signalling in the nuclear compartment by facilitating binding of phospho-SMAD3 to SMURF2 leading to SMURF2-mediated ubiquitin proteasomal degradation [48], L1 ORF1p alters PIN1 activity differentially, potentially by altering its ratio in the cellular compartments (decreased nuclear activity and increased cytoplasmic activity).

In combination, we propose that in HCC, aberrant L1 ORF1p expression leads to higher expression of cytoplasmic PIN1, which contributes towards dysregulation of a number of oncogenic pathways—including upregulation of TGFβ and NF-κB signalling—that promote tumour invasion and metastasis.

## Discussion

The development of personalised treatment for cancer requires the discovery of key drivers of tumour cell biology in diverse cancer types. The major contribution of activated L1 retrotransposons in cancer is attributed to their retrotransposition capability, leading to somatic mutagenesis and genomic rearrangements. The Influence of L1 on oncogenic pathways has been documented, but typically via insertional mutagenesis pathway [7, 19]. Our study integrates analysis of TCGA-HCC cohort RNAseq data and immunohistochemical analysis of an independent HCC cohort with in vitro modelling. The combination reveals a novel role for L1 ORF1p in HCC in the regulation of key oncogenic pathways promoting hepatocarcinogenesis—including TGFβ signalling and inflammatory response pathways independently of active retrotransposition. We have recently demonstrated activation of L1 in chronic hepatitis C (CHC) infected patients and that activated L1 can influence hepatocarcinogenesis beyond viral clearance [37]. The current study highlights the potential contribution of L1 expression in CHC patients towards hepatocarcinogenesis by alteration of key oncogenic pathways. However, the proposed model depicted in the graphical abstract awaits validation using in vivo models.

TGFβ signalling has both tumour promoter and suppressor activities, with a complexity that makes it hard to target therapeutically [55]. It is also a critical regulator of liver inflammation, being important not just in hepatocytes, but also in the surrounding environmental stellate and immune cells [56]. The impact of the TGFβ pathway is context-dependent. It functions as a tumour suppressor in premalignant or early-stage of cancers, when cell cycle arrest and apoptosis pathways are intact. However, in the later stages when tumour-promoting mechanisms that overcome cell cycle arrest are in play - such as *TP53* mutation—it can promote the progression of tumours, mediating cancer metastasis by inducing EMT [57, 58]. Thus, it is important to appreciate the “stage” and “cellular context”, when considering targeting the TGFβ pathway as a therapeutic intervention for HCC. Ongoing clinical trials for mono- and combination therapies of TGFβ inhibition with immunotherapy and radiotherapy include all comers, in the absence of predictive biomarkers for stratification. Predictive biomarkers defining TGFβ status are lacking but could potentially limit patient morbidity and costs. We propose increased L1 ORF1p expression in tumour cells as a candidate predictive biomarker worthy of consideration in ongoing clinical trials, of single agent TGFβ inhibitors, or those mono/combination approaches impacting the inflammatory/immune environment. The activation of L1 can be assessed by analysing L1 promoter hypomethylation in the circulating cell free DNA [12, 59], which is an added advantage when considering biomarker assay development.

Moreover, targeting the interaction of L1 ORF1p with host proteins or downstream signalling may have therapeutic value worthy of further exploration. This novel approach to alter TGFβ or other inflammatory oncogenic pathways would have the potential advantage of being ‘cancer specific’. In contrast to our observations, Zhu et al. have reported direct interaction of L1 ORF1p with SMAD4 leading to the suppression of the translocation of SMAD4 from the cytoplasm to the nucleus elicited by TGFβ [60]. This could be a potential negative feedback loop between L1 ORF1 overexpression and TGFβ signalling. We have identified cytoplasmic PIN1 upregulation consequent to overexpression of L1 ORF1p, with phosphorylation of L1 ORF1p at S18/S27 essential for the process of retrotransposition and PIN1 interaction [46]. Mutant L1 ORF1p (S18AS27A) also upregulating cytoplasmic PIN1 supports an indirect interaction, or a mechanism involving another L1 ORF1p motif. Unravelling the mechanism of PIN1 complex formation with L1 ORF1p may reveal novel therapeutic avenues. Recently, Napoletano et al. have demonstrated the role of PIN1 in the negative regulation of transposable elements including L1 transcripts in neurons during ageing or mechanical stress as PIN1 is essential for nuclear envelope integrity and heterochromatin maintenance [61]. However, in spite of high PIN1 expression in cancer cells, L1 expression is also upregulated, indicating dysregulation of other L1 regulatory mechanisms independent of PIN1 or reduced PIN1 nuclear activity in the presence of L1 ORF1.

Our observation of the upregulation of PIN1 in human HCC in situ, particularly in the cytoplasm in association with L1 ORF1p, is notable. In a number of other cancer types, cytoplasmic localisation of PIN1 has been associated with cancer aggressiveness and poor prognosis [49, 50]. Similarly, the contribution of cytoplasmic PIN1 to HCC aggressiveness merits further investigation. PIN1 influences various transcription factors mechanistically, influencing nucleocytoplasmic shutting, protein stability and altering binding partners [62]. For example, PIN1 inhibits p65 (a NF-κB subunit) binding to IκBα and enhances p65 nuclear localisation [54]. PIN1 downregulates TGFβ signalling in the nuclear compartment by facilitating binding of phospho-SMAD3 to SMURF2 leading to SMURF2-mediated ubiquitin proteasomal degradation [48], with our study showing this activity reduced in cells overexpressing L1 ORF1p. Hence, forming a complex with L1 ORF1p would potentially alter the interacting partners of PIN1 and its influence on oncogenic signalling pathways. Targeting PIN1, lying at the heart of signalling pathways important for cancer initiation and development [63] may have value. Over the past two decades, several PIN1 inhibitors have been developed, exhibiting pre-clinical in vitro and in vivo activities against human cancers, including HCC [64]. In addition, the development of more potent and specific PIN1 inhibitors for clinical applications is an active area of research [65, 66]. Recently, Sulfopin is reported as a highly selective covalent PIN1 inhibitor, showing promise as an anticancer agent [67]. Our study indicates that targeting the complex formation of PIN1 with L1 ORF1p can be explored further for fine-tuning the functions of PIN1 interacting proteins leading to HCC therapeutics.

## Conclusions

L1-encoded L1 ORF1p is specifically overexpressed in HCC compared to surrounding non-tumour liver tissue or immune infiltrates and influences oncogenic pathways by interacting with cellular proteins. In summary, we draw attention to the L1 ORF1p mediated cytoplasmic upregulation of PIN1 in human HCC, warranting its further exploration as a clinical predictive biomarker and novel anticancer drug development target for HCC.

## Supplementary information

Supplementary Information

## Data Availability

RNAseq data generated in the study are submitted to GEO under accession no. GSE126615 and GSE194251. Other materials generated in the study, such as plasmids, will be shared upon request.
