# Supplementary material for: Impact of retrotransposon protein L1 ORF1p expression on oncogenic pathways in hepatocellular carcinoma: the role of cytoplasmic PIN1 upregulation
Source: Br J Cancer. 2023 Jan 27;128(7):1236–48. doi: 10.1038/s41416-023-02154-9 (PMC10050422; doi:10.1038/s41416-023-02154-9)
Supplement: Supplementary file 1 — Supplementary Information [file 41416_2023_2154_MOESM1_ESM.docx]

**Supplementary Information**

**Methods**

**Cell proliferation by SRB assay**

1000 cells per well were seeded into a 96-well plates and incubated for the said time intervals. The cells were fixed using Carnoy’s fixative followed by Sulforhodamine B (SRB) assay[1].

**Cell proliferation by IncuCyte®**

500 cells per well were seeded into a 96-well plate and incubated in an IncuCyte® live cell imaging system. The plates were scanned every 6 hours and cell proliferation was evaluated using an IncuCyte® algorithm for cell confluence.

**Clonogenic assay**

4000 cells were seeded in a 10 cm petri dishes in triplicates and plates were incubated at 37˚C and 5% CO_2_ for 21 days. At the end of incubation, media was removed and dishes were washed with PBS and fixed with cold Carnoy fixative for 10 minutes. Plates were then washed with distilled water, and stained with 0.4% crystal violet (Sigma, C0775) for 30 minutes. Finally, plates were washed with distilled water and colony size and number were counted using an automatic colony counter (Oxford optronix COL COUNT).

**FACS**

Cells were fixed in 1% formalin and washed twice with perm/wash I buffer (BD bioscience, 557885). They were then stained with anti-L1 ORF1p (1: 4000, Merck MABC1152) for 1 hour (h) at room-temperature followed by anti-mouse-AF488 secondary antibody, (1:8000, Invitrogen, A11001) for 1 h at room-temperature. Finally, cells were washed and analysed on a BD FACS Calibur. Data analysis was carried out using Cyflogic software.

**Luciferase IHC**

Slides were dewaxed in xylene and hydrated in alcohol, citric saline antigen retrieval was performed and endogenous peroxidase activity was blocked using hydrogen peroxide. Inhibition of nonspecific binding was achieved using avidin/biotin blocking kit (Vector Laboratories) followed by incubation with pig serum. Slides were incubated at room temperature for one hour with anti-Luciferase Ab 1:2000. Slides were washed and incubated with biotininylated swine anti-rabbit Ab 1:200, followed by Vectastain ABC reagent. Luciferase positive cells were visualised by 3,3′-diaminobenzidine tetrahydrochloride (DAB) and counterstained with Gills II hematoxylin.

**References**

[1] Skehan P, Storeng R, Scudiero D, Monks A, McMahon J, Vistica D, et al. New colorimetric cytotoxicity assay for anticancer-drug screening. J Natl Cancer Inst 1990;82:1107-1112.

[2] Kong Y, Rose CM, Cass AA, Williams AG, Darwish M, Lianoglou S, et al. Transposable element expression in tumors is associated with immune infiltration and increased antigenicity. Nat Commun 2019;10:5228.

[3] Coulouarn C, Factor VM, Thorgeirsson SS. Transforming growth factor-beta gene expression signature in mouse hepatocytes predicts clinical outcome in human cancer. Hepatology 2008;47:2059-2067.

[4] Caruso S, Calatayud AL, Pilet J, La Bella T, Rekik S, Imbeaud S, et al. Analysis of Liver Cancer Cell Lines Identifies Agents With Likely Efficacy Against Hepatocellular Carcinoma and Markers of Response. Gastroenterology 2019;157:760-776.

**Supplementary Figure, Tables and Legends**


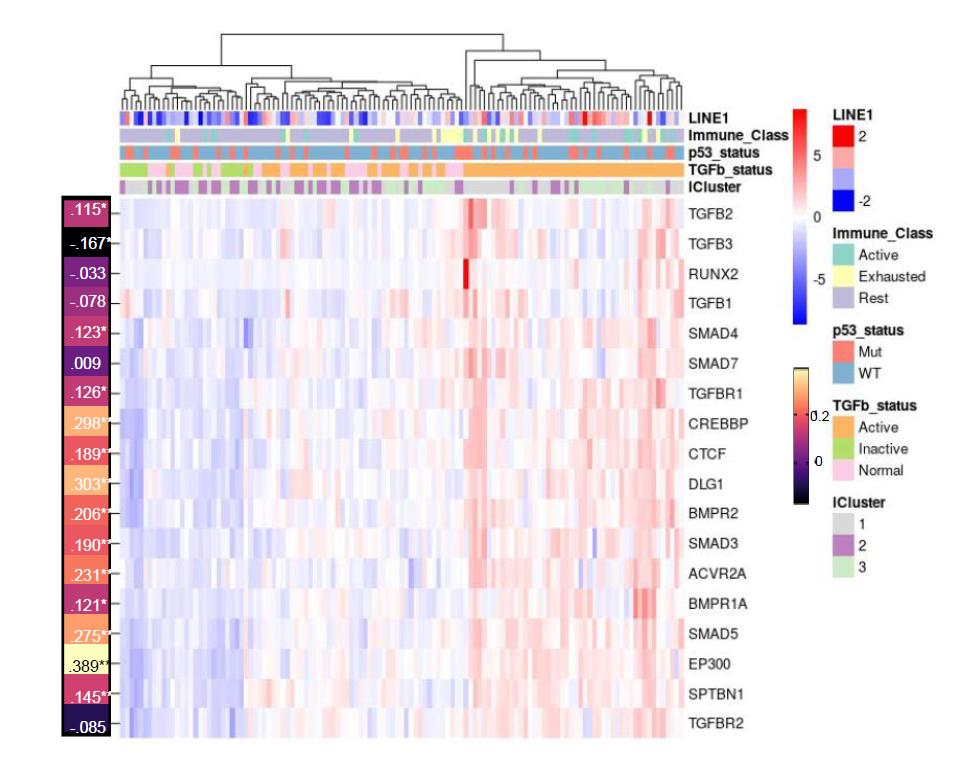


**Figure S1: Correlation between L1 expression and TGFβ superfamily genes.** Heatmap represents FPKM values of indicated genes for a subset (for which all the annotations were available) of TCGA-LIHC cohort samples (n = 123). LINE1 = L1 counts. Heatmap bar on the left hand side represents values of Spearman r correlation between the gene and L1 expression for all the samples of TCGA-LIHC cohort (n=372). * P < 0.05, ** P<0.01.


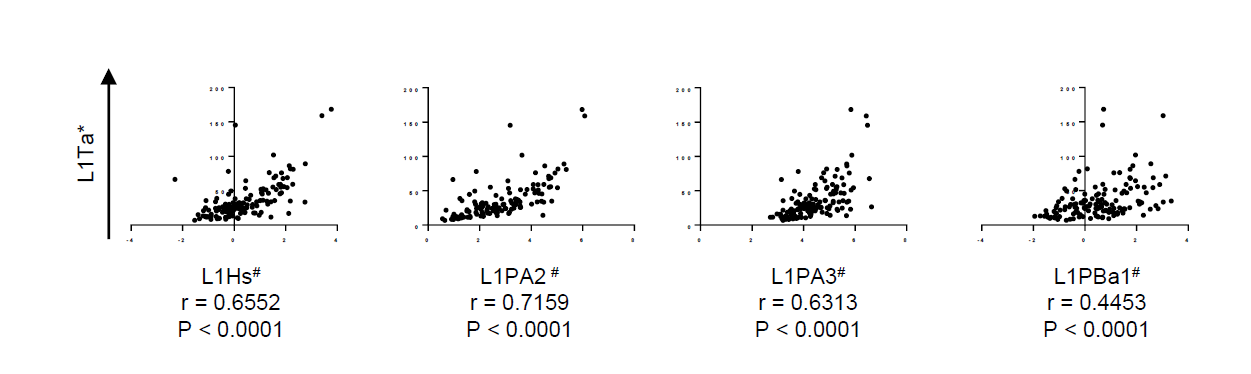


**Figure S2: Characterisation of L1 transcript expression in HCC.** Correlation graphs between L1 transcript counts obtained for L1-Ta sequence by in-house pipeline and L1 counts for indicated L1 subfamilies reported by using REdiscoverTE script[2].


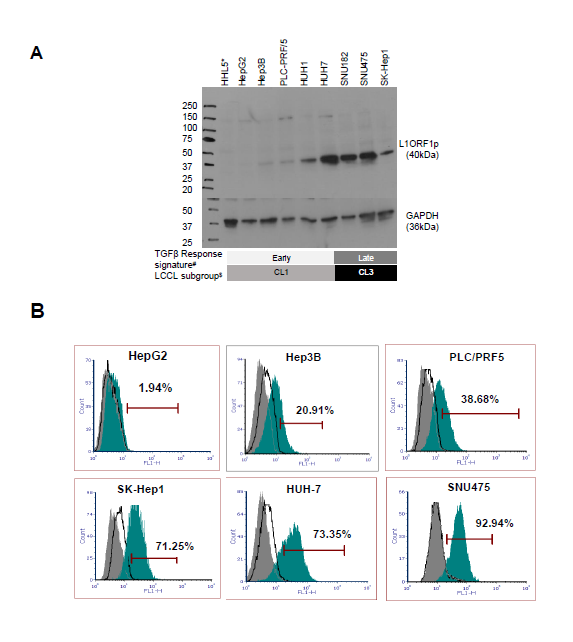


**Figure S3: L1 ORF1p expression in different liver cancer cell lines.** (A) Western blot of indicated cell lines showing L1 ORF1p expression in the whole cell lysates. GAPDH was used as a loading control. Bottom panel represents previous classifications of these cell lines based on transcriptomic data. # Coulouarn et al[3] classified the cell lines based on their response to TGFβ stimulus. $ Caruso et al[4] classed 34 liver cancer cell lines (excluding SK-Hep1) into 3 groups (CL1-CL3) representing an increase in epithelial to mesenchymal signature from CL1 to CL3. *HHL5 cells are immortalised human non-cancerous hepatocytes used here as a negative control for L1 ORF1p and are not included in any of the previous classifications. (B) Grey shaded peaks represent unstained control, black lines represent secondary staining only (anti-mouse-AF488; no primary control) and green shaded peaks represent L1 ORF1p + anti-mouse-AF488 stained cells. L1 ORF1p expression was determined by setting the gate at 0.5% of secondary only cells.


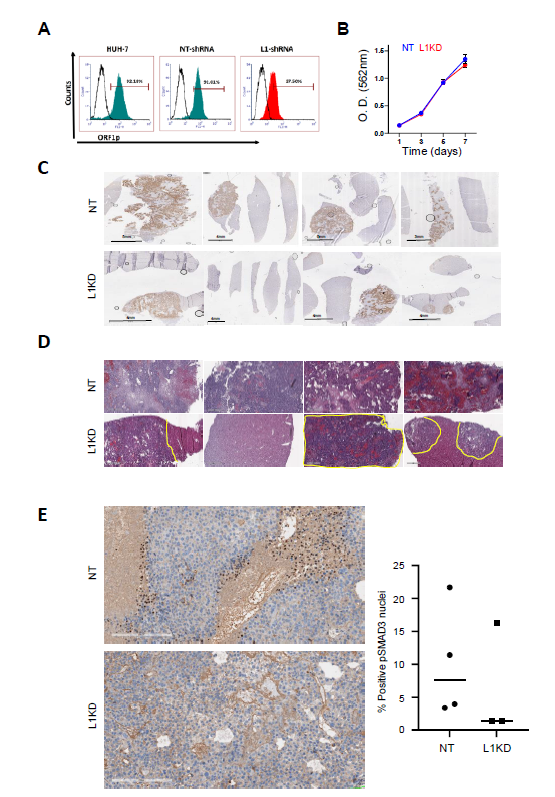


**Figure S4: Influence of L1 knockdown on *in-vitro* proliferation and *in-vivo* tumorigenesis in Huh7 cells.** (A) FACS plot confirming knockdown of L1 ORF1p. Black lines represent secondary only control and shaded peaks represent L1 ORF1p + anti-mouse-AF488 stained cells. (B) Representative growth curve of Huh7-NT and Huh7-L1KD cells assessed by SRB assay. Graph represents mean ± standard error of 3 technical replicates. (C) Luciferase IHC images of tumour developed in the liver and background liver slices of mouse injected with Huh7-NT or Huh7-L1KD cells. (D) H & E staining of tumour sections showing large necrotic areas in the NT group. L1KD group had smaller tumours (marked with yellow line) and rest is background mouse liver tissue. (E) Representative IHC images for pSMAD3 in the xenograft tissues from mice injected with Huh7-NT or Huh7-L1KD cells. Graph on the right represents quantification of pSMAD3 positive nuclei in the two groups.


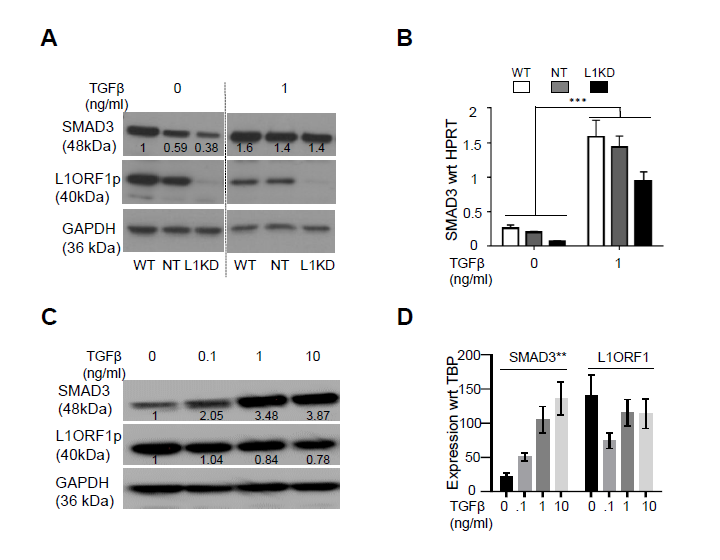


**Figure S5: Influence of TGFβ on SMAD3 and L1 ORF1p in Huh7 cells.** (A) Western blot analysis of indicated cells showing influence of TGFβ treatment on SMAD3 level. GAPDH is used as a loading control. Numbers on the blot represents fold change expression with respect to WT cells normalised using GAPDH. (B) RT-qPCR analysis of indicated cells to measure *SMAD3* with and without TGFβ stimulation for 72 h. *HPRT* was used as a housekeeping gene. Data shows representative experiment of 3 independent repeats. *** P < 0.001, 2way-ANOVA. (C) Western blot analysis of Huh7 cells showing influence of TGFβ treatment on L1 ORF1p and SMAD3 levels. GAPDH is used as a loading control. Numbers on the blot represents fold change expression with respect to WT cells normalised using GAPDH. (D) RT-qPCR analysis of Huh7 cells to measure *L1 ORF1* and *SMAD3* with and without TGFβ stimulation for 48 h. *TBP* was used as a housekeeping gene. Data shows representative experiment of 3 independent repeats. ** P < 0.01, one-way ANOVA.


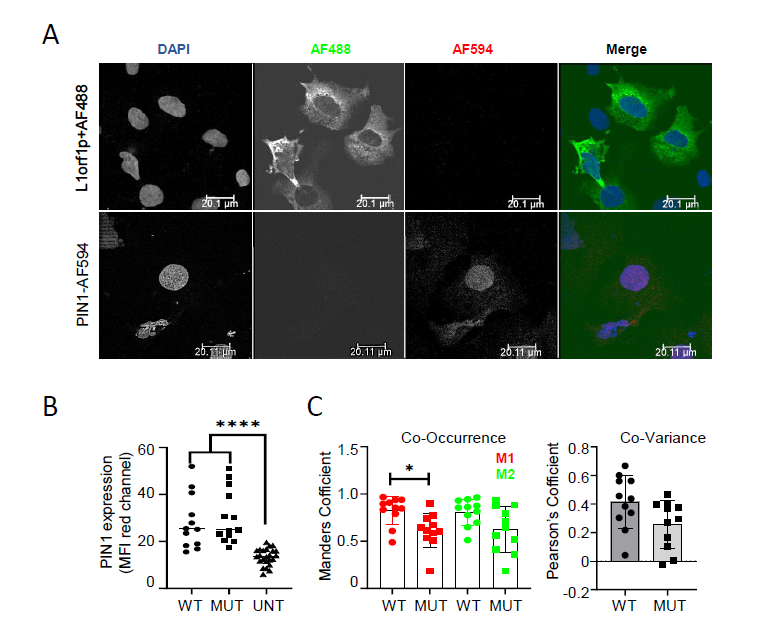


**Figure S6: Controls and quantification of immunocytochemical (ICC) images of Hep3B cells co-stained for L1 ORF1p and Pin1.** (A) ICC images showing single antibody staining to control channels bleed-through for dual staining in Hep3B cells transfected with L1 ORF1 expression plasmid (B) Graph representing quantification of mean fluorescent intensity (MFI) of red channel (AF594, representative of Pin1 expression) of individual cells expressing either WT or mutant (MUT) L1 ORF1p or adjoining non-transfected (UNT) cells. **** P < 0.0001, one-way ANOVA with multiple comparisions. (C) Co-localisation analysis of Pin1 and L1 ORF1p (WT or MUT) - graphs represent co-occurrence as Manders coefficient where M1 = proportion of the intensity from the green channel that co-localises with the red channel and M2 = proportion of the intensity from the red channel that co-localises with the green channel; Co-variance of the 2 channels is represented as Pearson’s coefficient. * P < 0.05, Mann-Whitney test.


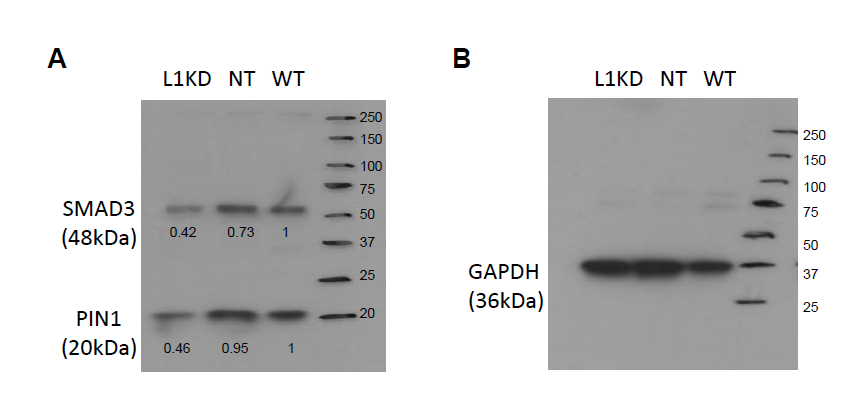


**Figure S7: Influence of L1 ORF1 knockdown on PIN1 in Huh7 cells.** (A) Western blot image showing expression of PIN1 and SMAD3 in whole cell lysates of the indicated cell lines. The blot was first probed with anti-PIN1 followed by anti-SMAD3 antibody without stripping. Numbers on the blot represents fold change expression with respect to WT cells normalised using GAPDH. (B) Western blot image showing GAPDH as a loading control for the blot in S7A. The blot was stripped and then reprobed with anti-GAPDH antibody.


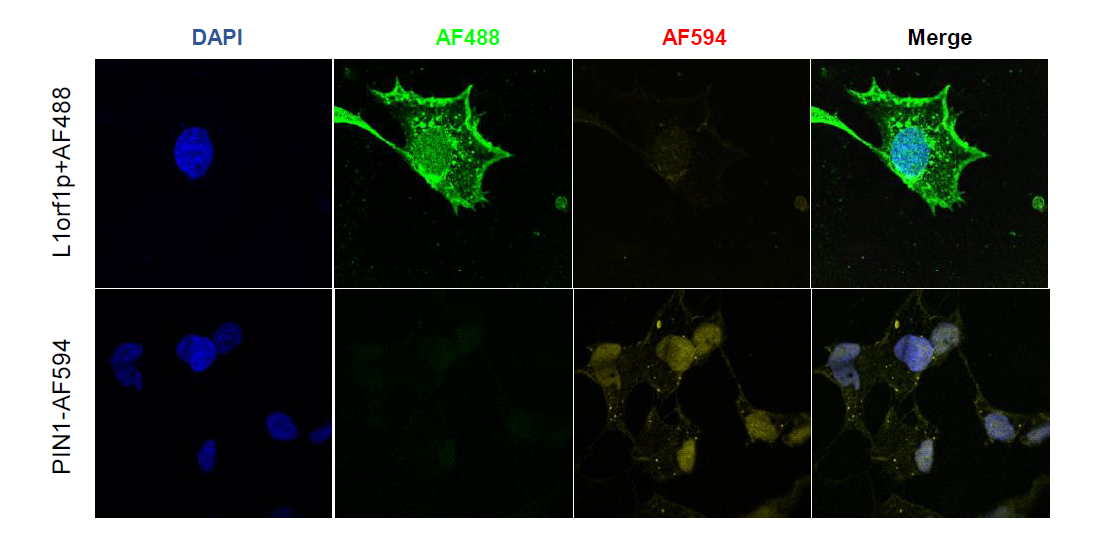


**Figure S8:** Immunocytochemical images showing single antibody staining to control channels bleed-through for dual staining in Hep3B-DOX-L1 ORF1 cells induced with Dox for 48 h.


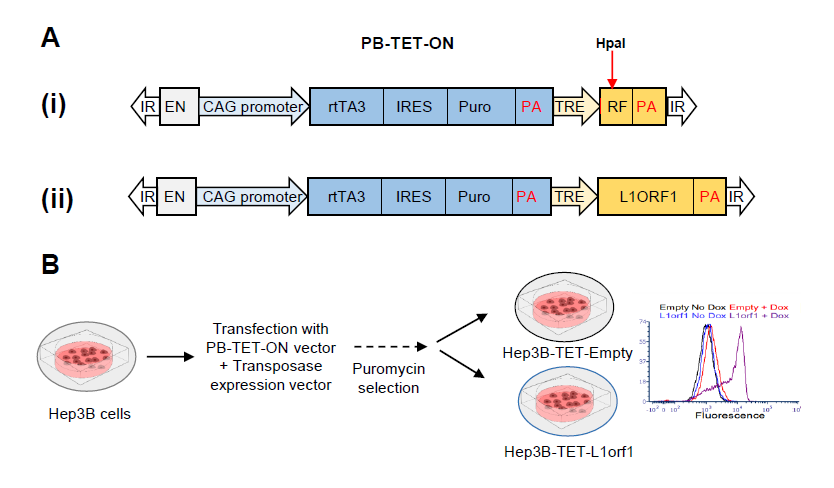


**Figure S9: Generation of doxycycline (DOX) inducible L1 ORF1 Hep3B and control cells.** (A) Schematics of the piggyback vector used to generate the empty vector control and L1 ORF1 cells. HpaI site was used to clone codon optimised L1 ORF1 sequence. (B) Schematics representing derivation of indicated stable cell lines.


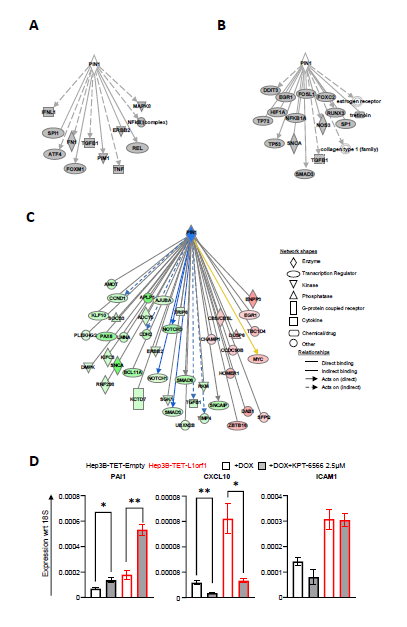


**Figure S10: IPA generated PIN1 interaction network.** (A-B) PIN1 interacts with several upstream regulators of differentially expressed genes identified in Hep3B-DOX-L1 ORF1 versus Hep3B-DOX-Empty (A) and Huh7-L1KD versus Huh7-NT (B). (C) Interaction map of PIN1 with differentially expressed genes in Huh7-L1KD versus Huh7-NT cells. Shades of green represent downregulated and red represents upregulated genes. Blue colour of PIN1 indicates IPA predicts inhibition of PIN1 activity. Network shapes and relationships shown in ‘C’ are for ‘A’ and ‘B’ as well. (D) Graphs representing RT-qPCR results of indicated transcripts in Hep3B-DOX-Empty and Hep3B-DOX-L1 ORF1 cells upon Dox induction (1µg/ml for 48 h) alone or in combination with KPT-6566 (Pin1 inhibitor, 2.5µM) for the last 24 h. 18S was used as a normalisation control. Please note, Hep3B-DOX-L1 ORF1 were more sensitive to KPT-6566 than Hep3B-DOX-Empty cells. At 10µM, effect of KPT-6566 was enhanced in Hep3B-DOX-Empty than 2.5µM however was toxic for Hep3B-DOX-L1 ORF1 cells. * P < 0.05, ** P < 0.01, unpaired t-test.

**Table S1. Descriptive table of clinical and histological features of the TCGA-LIHC cohort and their associations with HCC L1 transcripts status**

| **Clinical Features** | | **N (%)** | | | **p-value** |
| --- | --- | --- | --- | --- | --- |
|  |  | **Total 192 (100)** | **L1-low 81 (42)** | **L1-high 111(58)** |  |
| Age | Median | 62 [20-90] | 61 [20-90] | 62 [20-85] | ns |
| Gender | Male/Female (F%) | 126/66 (34) | 56/25 (31) | 70/41 (37) | ns |
| Ethnicity | Caucasian/Asian/African-American/Other | 113/55/14/2 | 49/21/2/2 | 64/34/12/0 | ns |
| Risk Factor | Viral/NAFLD-ALD/Other/No known risk | 55/60/13/56 | 25/23/5/25 | 30/37/8/31 | ns |
| Obese or Overweight | No/Yes (Y%) | 79/84 (52) | 41/29 (41) | 43/50 (54) | ns |
| Cirrhosis | No/Yes (Y%) | 139/47 (25) | 60/18 (23) | 79/29 (27) | ns |
| **Tumor and Histopathologic Features** | | | | | |
| TNM Stage | I/II/III/IV (>I%) | 74/43/58/5 (59) | 42/14/17/4 (45) | 32/29/41/1 (69) | 0.003 |
| AFP (>300 ng/ml) | No/Yes (Y%) | 103/31 (22) | 46/8 (15) | 57/23 (29) | 0.046 |
| Vascular Invasion | No/Yes (Y%) | 105/53 (34) | 51/16 (24) | 54/37 (41) | 0.01 |
| Grade Well Differentiated | No/Yes (Y%) | 132/47 (54) | 35/25 (42) | 68/22 (24) | 0.024 |
| Growth pattern | Solid/Trabecular/Macrotrabecular/Pseudoacinar | 77/72/19/15 | 33/26/7/8 | 41/45/12/7 | ns |
| IT fibrosis | No/Yes (Y%) | 140/43 (23) | 50/24 (32) | 87/18 (17) | 0.017 |
| IT cholestasis | No/Yes (Y%) | 134/49 (27) | 48/27 (36) | 84/21 (20) | 0.017 |
| IT inflammation | No/Yes (Y%) | 143/40 (22) | 60/14 (19) | 81/24 (23) | ns |
| Ballooned hepatocytes | No /Yes (Y%) | 144/39 (22) | 57/17 (23) | 86/19 (18) | ns |

NOTE: Patients are grouped into 2 categories based on their L1 transcript expression in HCC (L1-low = L1 expression equivalent to L1 expression in non-tumour liver, L1-high = L1 expression higher than non-tumour liver (L1 normalised count >25)). Categorical and continuous datasets were compared with Pearson’s chi-square (Fishers Exact for groups with <5 cases) and Mann-Whitney U tests respectively; ns = not significant.

**Table S2: Descriptive table of clinical and histological features of the patient cohort used for immunohistochemical (IHC) analysis of L1orf1p and their associations with HCC L1orf1p status**

| **Clinical Features** | | **N (%)** | | | **p-value** |
| --- | --- | --- | --- | --- | --- |
|  |  | **Total 48 (100)** | **L1-low 21 (44)** | **L1-high 27 (56)** |  |
| Age | Median [range] | 71 [49-85] | 71 [55-85] | 70.8 [49-80] | ns |
| Gender | Male/Female (F%) | 38/10 (21) | 17/04 (19) | 21/06 (22) | ns |
| Risk Factor | NAFLD/ALD/Viral/Others/No known risk | 24/4/2/6/12 | 13/0/2/2/4 | 11/4/0/4/8 | ns |
| Obese or Overweight | No/Yes (Y%) | 13/21 (62) | 4/10 (71) | 9/11 (55) | ns |
| Diabetes | No/Yes (Y%) | 17/29 (63) | 5/14 (74) | 12/15 (56) | ns |
| Cirrhosis | No/Yes (Y%) | 28/20 (42) | 11/10 (48) | 17/10 (37) | ns |
| **Tumour and Histopathologic Features** | | | | | |
| Tumour number | Median [range] | 1.64 [1-20] | 1.77 [1-10] | 1.55 [1-20] | ns |
| Tumour size | Median [range] | 7.30 [1.3-22] | 6.20 [2.7-22] | 8.50 [1.3-18] | ns |
| PVT | No/Yes (Y%) | 34/14 (29) | 15/6 (29) | 19/8 (29) | ns |
| TNM Stage | I/II/III/IV (>I%) | 18/6/16/8 (63) | 6/2/7/6 (71) | 12/4/9/2 (56) | ns |
| Grade Well Differentiated | No/Yes (Y%) | 27/19 (40) | 9/12 (57) | 18/7 (27) | 0.046 |
| BCLC Stage | A/B/C/D | 8/9/29/2 | 4/4/12/1 | 4/5/17/1 | ns |
| **Survival post biopsy Months median** | | | | | |
| All patients | N = 48 | 14.73 (100) | 20.05 (42) | 14.60 (58) | ns |
| Treated patients | N = 39 | 19.9 (100) | 29.23 (38) | 17.72 (62) | ns |
| TACE treated | N = 24 | 14.67 (100) | 24.29 (38) | 12.90 (62) | 0.017 |

Patients are grouped into 2 categories based on their HCC L1orf1p IHC. Categorical and continuous datasets were compared with Pearson’s chi-square (Fishers Exact for groups with <5 cases) and Mann-Whitney U tests respectively; ns = not significant. Since pilot staining of 10 samples revealed 100% positivity for L1orf1p, total 50 samples were retrieved from our biobank for this study. Due to poor tissue quality 2 samples were excluded.

**Table S3: Hallmark gene sets enriched in (A) Huh7-NT cells when compared to Huh7-L1KD cells, (B) Hep3B-DOX-L1orf1 cells when compared to Hep3B-DOX-Empty cells, by GSEA.**

**A**

| **NAME** | **SIZE** | **ES** | **NES** | **NOM p-val** | **FDR q-val** | **FWER p-val** | **RANK AT MAX** |
| --- | --- | --- | --- | --- | --- | --- | --- |
| HALLMARK_ANGIOGENESIS | 26 | -0.69081 | -2.5698 | 0 | 0 | 0 | 1274 |
| HALLMARK_EPITHELIAL_MESENCHYMAL_TRANSITION | 139 | -0.46928 | -2.38035 | 0 | 0 | 0 | 1719 |
| HALLMARK_HYPOXIA | 166 | -0.43354 | -2.27861 | 0 | 0 | 0 | 1452 |
| HALLMARK_MYOGENESIS | 122 | -0.44465 | -2.23065 | 0 | 0 | 0 | 1240 |
| HALLMARK_UV_RESPONSE_DN | 130 | -0.3209 | -1.6059 | 0.005917 | 0.014884 | 0.054 | 1277 |
| HALLMARK_APICAL_SURFACE | 28 | -0.41018 | -1.5184 | 0.027875 | 0.024448 | 0.1 | 890 |
| HALLMARK_APICAL_JUNCTION | 137 | -0.29365 | -1.50679 | 0 | 0.023622 | 0.112 | 1823 |
| HALLMARK_TGF_BETA_SIGNALING | 50 | -0.35233 | -1.50568 | 0.019231 | 0.021082 | 0.114 | 1898 |
| HALLMARK_HEDGEHOG_SIGNALING | 23 | -0.42242 | -1.47534 | 0.056667 | 0.024625 | 0.151 | 1449 |
| HALLMARK_KRAS_SIGNALING_DN | 76 | -0.29416 | -1.37129 | 0.043902 | 0.050214 | 0.297 | 1149 |
| HALLMARK_NOTCH_SIGNALING | 27 | -0.37509 | -1.36884 | 0.099644 | 0.046863 | 0.305 | 843 |
| HALLMARK_PANCREAS_BETA_CELLS | 17 | -0.40319 | -1.27888 | 0.182759 | 0.079054 | 0.496 | 729 |
| HALLMARK_ESTROGEN_RESPONSE_EARLY | 155 | -0.24298 | -1.27127 | 0.058394 | 0.075638 | 0.505 | 1053 |

**B**

| **NAME** | **SIZE** | **ES** | **NES** | **NOM p-val** | **FDR q-val** | **FWER p-val** | **RANK AT MAX** |
| --- | --- | --- | --- | --- | --- | --- | --- |
| HALLMARK_PANCREAS_BETA_CELLS | 40 | 0.653542 | 1.76427 | 0 | 0.003379 | 0.002 | 1572 |
| HALLMARK_XENOBIOTIC_METABOLISM | 198 | 0.483384 | 1.660387 | 0 | 0.009102 | 0.011 | 2978 |
| HALLMARK_MYC_TARGETS_V2 | 58 | 0.566445 | 1.627448 | 0.004184 | 0.012065 | 0.02 | 7037 |
| HALLMARK_UNFOLDED_PROTEIN_RESPONSE | 110 | 0.506136 | 1.612716 | 0 | 0.009825 | 0.022 | 3348 |
| HALLMARK_KRAS_SIGNALING_UP | 199 | 0.467734 | 1.606431 | 0 | 0.00786 | 0.022 | 2815 |
| HALLMARK_COAGULATION | 138 | 0.458534 | 1.508028 | 0.002283 | 0.028865 | 0.098 | 2486 |
| HALLMARK_TNFA_SIGNALING_VIA_NFKB | 199 | 0.428757 | 1.470814 | 0 | 0.039968 | 0.151 | 3012 |
| HALLMARK_BILE_ACID_METABOLISM | 112 | 0.456568 | 1.450409 | 0.014056 | 0.042346 | 0.179 | 2962 |
| HALLMARK_INTERFERON_ALPHA_RESPONSE | 96 | 0.449591 | 1.408651 | 0.013015 | 0.05668 | 0.267 | 2317 |
| HALLMARK_IL6_JAK_STAT3_SIGNALING | 87 | 0.452241 | 1.406219 | 0.023605 | 0.052762 | 0.273 | 2552 |
| HALLMARK_UV_RESPONSE_UP | 156 | 0.418315 | 1.396636 | 0.008602 | 0.051642 | 0.289 | 3872 |
| HALLMARK_MTORC1_SIGNALING | 197 | 0.40537 | 1.386917 | 0.004376 | 0.052589 | 0.312 | 6528 |
| HALLMARK_COMPLEMENT | 200 | 0.400253 | 1.381864 | 0.002247 | 0.051601 | 0.328 | 3349 |
| HALLMARK_INTERFERON_GAMMA_RESPONSE | 198 | 0.400613 | 1.373895 | 0.004425 | 0.053011 | 0.355 | 3184 |
| HALLMARK_TGF_BETA_SIGNALING | 54 | 0.444075 | 1.291631 | 0.088553 | 0.110234 | 0.624 | 3130 |
| HALLMARK_HEME_METABOLISM | 195 | 0.368112 | 1.271589 | 0.025404 | 0.12328 | 0.682 | 3282 |
| HALLMARK_IL2_STAT5_SIGNALING | 199 | 0.362162 | 1.246951 | 0.024887 | 0.147203 | 0.757 | 2918 |
| HALLMARK_EPITHELIAL_MESENCHYMAL_TRANSITION | 197 | 0.360342 | 1.239035 | 0.050114 | 0.149948 | 0.781 | 3307 |
| HALLMARK_ALLOGRAFT_REJECTION | 196 | 0.355486 | 1.22872 | 0.045238 | 0.156531 | 0.816 | 3125 |
| HALLMARK_HEDGEHOG_SIGNALING | 36 | 0.445917 | 1.193615 | 0.190578 | 0.198737 | 0.9 | 4586 |
| HALLMARK_WNT_BETA_CATENIN_SIGNALING | 42 | 0.436939 | 1.192991 | 0.190855 | 0.190281 | 0.901 | 2790 |
| HALLMARK_ESTROGEN_RESPONSE_LATE | 197 | 0.34137 | 1.188695 | 0.079498 | 0.189074 | 0.907 | 3636 |
| HALLMARK_INFLAMMATORY_RESPONSE | 200 | 0.336577 | 1.164995 | 0.107551 | 0.220183 | 0.945 | 5047 |
| HALLMARK_FATTY_ACID_METABOLISM | 157 | 0.335304 | 1.120783 | 0.180365 | 0.301587 | 0.987 | 3532 |
| HALLMARK_MYC_TARGETS_V1 | 196 | 0.322825 | 1.113689 | 0.17094 | 0.305905 | 0.991 | 8423 |
| HALLMARK_HYPOXIA | 197 | 0.318865 | 1.096315 | 0.184486 | 0.333928 | 0.995 | 3951 |
| HALLMARK_P53_PATHWAY | 196 | 0.317698 | 1.093491 | 0.181193 | 0.328782 | 0.997 | 3093 |
| HALLMARK_UV_RESPONSE_DN | 142 | 0.321607 | 1.06178 | 0.302575 | 0.396457 | 1 | 1683 |
| HALLMARK_E2F_TARGETS | 198 | 0.30991 | 1.05483 | 0.298405 | 0.400812 | 1 | 6411 |
| HALLMARK_PI3K_AKT_MTOR_SIGNALING | 105 | 0.320744 | 1.032297 | 0.334831 | 0.452296 | 1 | 4037 |
| HALLMARK_G2M_CHECKPOINT | 196 | 0.290428 | 0.996298 | 0.452703 | 0.553986 | 1 | 6641 |
| HALLMARK_APICAL_SURFACE | 44 | 0.358179 | 0.987498 | 0.474836 | 0.562345 | 1 | 3158 |
| HALLMARK_ADIPOGENESIS | 198 | 0.287647 | 0.984759 | 0.464286 | 0.552947 | 1 | 3766 |
| HALLMARK_GLYCOLYSIS | 198 | 0.279644 | 0.965661 | 0.536131 | 0.596746 | 1 | 3717 |
| HALLMARK_ANDROGEN_RESPONSE | 99 | 0.306637 | 0.956725 | 0.547046 | 0.607917 | 1 | 3579 |
| HALLMARK_ANGIOGENESIS | 36 | 0.358584 | 0.937758 | 0.567347 | 0.654537 | 1 | 1282 |
| HALLMARK_REACTIVE_OXYGEN_SPECIES_PATHWAY | 49 | 0.279936 | 0.781528 | 0.885835 | 0.990006 | 1 | 7307 |
| HALLMARK_DNA_REPAIR | 149 | 0.209164 | 0.695255 | 1 | 0.994556 | 1 | 5978 |

**Table S4: Details of antibodies utilised in the study**

| **Immunohistochemistry by Ventana Discovery XT system** | | | | | | | | | |
| --- | --- | --- | --- | --- | --- | --- | --- | --- | --- |
| **Target** | **Producer** | | **Identifier** | | **Host species** | | **Dilution** | **De-masking reagent** | |
| L1orf1p | K Burns lab | | Clone 4H1 | | Mouse mAb | | 1:1000 | Discovery CC1 (Roche 06414575001 (950-500) | |
| pSMAD3 | Santa Cruz | | sc-517575 | | Mouse mAb | | 1:50 |  |  |
| Pin1 | Santa Cruz | | sc-46660 | | Mouse mAb | | 1:50 | CC2 (Roche 0527988001 (950-123) | |
| **Immunocytochemistry** | | | | | | | | |  |
| **Target** | | **Producer** | | **Identifier** | | **Host species** | | **Dilution** | **Incubation** |
| L1orf1p | | Merck | | MABC1152 (clone 4H1) | | Mouse | | 1:200 | Overnight 4°C |
| Pin1 | | Santa Cruz | | sc-46660 | | Mouse | | 1:250 | Overnight 4°C |
| Pin1 | | Santa Cruz | | sc-46660 AF594 | | Mouse | | 1:250 | 1h Room Temperature |
| pSMAD3 | | Santa Cruz | | sc-517575 | | Mouse | | 1:50 | Overnight 4°C |
| anti-mouse-AF647  (secondary antibody) | | Abcam | | Ab150115 | | Goat | | 1:2000 | 1h Room Temperature |
| anti-mouse-AF488 (secondary antibody) | | Invitrogen | | A11001 | | Goat | | 1:2000 | 1h Room Temperature |
| **Western Blotting** | | | | | | | | |  |
| **Target** | | **Producer** | | **Identifier** | | **Clonality/host species** | | **Dilution** | |
| L1orf1p | | Merck | | MABC1152 (clone 4H1) | | Mouse | | 1:1000 | |
| SMAD3 | | Abcam | | ab28379 | | Rabbit | | 1:1000 | |
| Pin1 | | Santa Cruz | | SC-46660 | | Mouse | | 1:1000 | |
| GAPDH | | Sigma | | G9545 | | Rabbit | | 1:10000 | |
| Anti-Mouse IgG-HRP | | DAKO | | P0447 | | Goat | | 1:2000 | |
| Anti-Rabbit IgG-HRP | | DAKO | | P0448 | | Goat | | 1:2000 | |

**Table S5: Sequences of primers used in the study for RT-qPCR**

| **Target** | **Sequence** |
| --- | --- |
| 18s_F | GTAACCCGTTGAACCCCATT |
| 18s_R | CCATCCAATCGGTAGTAGCG |
| TBP_F | GCAAGGGTTTCTGGTTTGCC |
| TBP_R | GGGTCAGTCCAGTGCCATAA |
| HPRT_F | TTGCTTTCCTTGGTCAGGCA |
| HPRT_R | ATCCAACACTTCGTGGGGTC |
| SMAD3_F | GAGGAGAAATGGTGCGAGAA |
| SMAD3_R | GCGGCAGTAGATGACATGAG |
| PAI1_F | CACAAATCAGACGGCAGCACT |
| PAI1_R | CATCGGGCGTGGTGAACTC |
| L1orf1_F | AGTGCTTAAAGGAGCTGATGG |
| L1orf1_R | GCTGATACCCTTTCTTCCAGTT |
| CXCL10_F | AAGTGGCATTCAAGGAGTACCT |
| CXCL10_R | ACACGTGGACAAAATTGGCT |
| ICAM1_F | AGCTTCGTGTCCTGTATGGC |
| ICAM1_R | TTTTCTGGCCACGTCCAGTT |
| IFIH1_F | AGATGCAACCAGAGAAGATCCA |
| IFIH1_R | TGGCCCATTGTTCATAGGGT |
